# Harmine Hydrochloride Mediates the Induction of G2/M Cell Cycle Arrest in Breast Cancer Cells by Regulating the MAPKs and AKT/FOXO3a Signaling Pathways

**DOI:** 10.3390/molecules26216714

**Published:** 2021-11-05

**Authors:** Chae Won Ock, Gi Dae Kim

**Affiliations:** 1 College of Pharmacy, Natural Products Research Institute, Seoul National University, Seoul 08826, Korea; ockchae1@naver.com; 2 Department of Food and Nutrition, Kyungnam University, Changwon 51767, Korea

**Keywords:** harmine hydrochloride, G2/M cell cycle arrest, AKT/FOXO3a

## Abstract

Breast cancer (BC) is one of the most common causes of death among women worldwide. Recently, interest in novel approaches for BC has increased by developing new drugs derived from natural products with reduced side effects. This study aimed to treat BC cells with harmine hydrochloride (HMH) to identify its anticancer effects and mechanisms. HMH treatment suppressed cell growth, migration, invasion, and colony formation in MCF-7 and MDA-MB-231 cells, regardless of the hormone signaling. It also reduced the phosphorylation of PI3K, AKT, and mTOR and increased FOXO3a expression. Additionally, HMH treatment increased p38 phosphorylation in MCF-7 cells and activated c-Jun N-terminal kinase (JNK) phosphorylation in MDA-MB-231 cells in a dose-dependent manner, where activated p38 and JNK increased FOXO3a expression. Activated FOXO3a increased the expression of p53, p21, and their downstream proteins, including p-cdc25, p-cdc2, and cyclin B1, to induce G2/M cell cycle arrest. Furthermore, HMH inhibited the PI3K/AKT/mTOR pathway by significantly reducing p-AKT expression in combination with LY294002, an AKT inhibitor. These results indicate that mitogen-activated protein kinases (MAPKs) and AKT/FOXO3a signaling pathways mediate the induction of cell cycle arrest following HMH treatment. Therefore, HMH could be a potential active compound for anticancer bioactivity in BC cells.

## 1. Introduction

Breast cancer (BC) is one of the most common causes of death in women worldwide and is closely associated with hormones and hormone receptors [1]. Estrogen receptor (ER)-positive BC accounts for 80% of total BC, and the remaining 20% are human epidermal growth factor receptor 2 (HER2)-positive BC. Triple-negative breast cancer (TNBC), which lacks ER α, progesterone receptors, and HER2, poses a high risk of relapse and metastasis and has a low survival rate after development [2]. Although the prognosis of patients with BC has improved with treatment, lifestyle modification, early detection, and mastectomy [3], the existing chemotherapeutics have unsatisfactory effectiveness, with some patients showing resistance to chemotherapy drugs [4]. There are complex and diverse risk factors for BC, and the molecular mechanisms underlying pathogenesis have not yet been elucidated. Thus, interest in novel approaches, such as developing new drugs derived from natural products effective in BC treatment with reduced side effects, is increasing [5].

Harmine hydrochloride (HMH) is a derivative of harmine. It is a combination of beta-carboline alkaloid isolated from the seeds of *Peganum harmala*, a traditional food and drug used in the Middle East, Central Asia, and South America, and hydrochloride to increase water solubility and bioavailability [6,7]. Studies have reported that harmine suppresses cancer progression by regulating cyclooxygenase-2 expression to inhibit stomach cancer cell proliferation [8]. It upregulates p21 and p27, followed by regulation of the G1/S phase cyclin-dependent kinases (CDKs) and cyclins, and induces G1 arrest [9]. In addition, other studies have reported that HMH suppresses stomach cancer cell proliferation by regulating its invasion and migration and inducing apoptosis [10]. In addition, HMH induces apoptosis in glioblastoma cells by inhibiting Akt phosphorylation and hepatoblastoma HepG2 cells through G2 cell cycle arrest and the mitochondrial pathway [11,12]. However, studies on its activation and mechanisms in BC cells are lacking.

Mitogen-activated protein kinases (MAPKs) include c-Jun N-terminal kinase (JNK) and p38 MAPK, which regulate a variety of cell activities, including proliferation, differentiation, apoptosis, inflammation, and innate immunity [13,14]. The JNK and p38 MAPK signaling pathways are activated by various types of cellular stress, including oxidation, differentiation, apoptosis, genotoxicity, and osmotic stress and microbial components, bacterial lipopolysaccharides, and proinflammatory cytokines TNF-α and IL-1β [15,16,17]. JNK and p38 are involved in the activation of FOXO3a in response to oxidative stress [18,19,20].

The PI3K/AKT/mTOR pathway is involved in regulating physiological processes, including cellular proliferation, adhesion, and motility. This pathway is also associated with multiple pathological processes, such as colorectal cancer [21], BC [22], liver cancer [23], and pancreatic cancer [24]. Previous studies have shown that increased AKT activation could promote proliferation and treatment resistance in BC cells [25,26]. FOXO3a is a downstream target of the PI3K/AKT pathway, which is correlated with AKT phosphorylation [27] and promotes the expression of genes, such as p21, p27, and cyclin D, which induce cell cycle arrest and suppress cancer cell proliferation [28,29]. In addition to DNA damage-mediated apoptosis, FOXO3a plays a vital role in initiating cell cycle arrest [30]. FOXO3a is an important tumor suppressor that is rarely expressed in many cases of BC. Its expression increases upon treatment with multiple anticancer drugs, implying its potential as a target for BC treatment [31].

**Figure 1:**
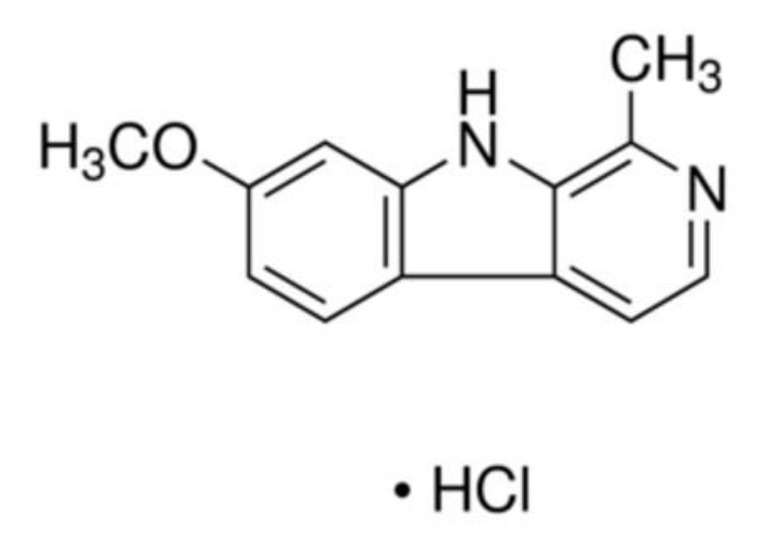
Chemical structure of harmine hydrochloride.

In this study, the anticancer effects and their mechanisms were investigated by treating BC cell lines, including ER-positive MCF-7 cells and TNBC MDA-MB-231 cells, with HMH (Figure 1), which has the same characteristics as harmine but with higher stability and absorption into tissues as a water-soluble compound.

## 2. Results

### 2.1. HMH Suppresses Cell Proliferation in BC Cells

**Figure 2:**
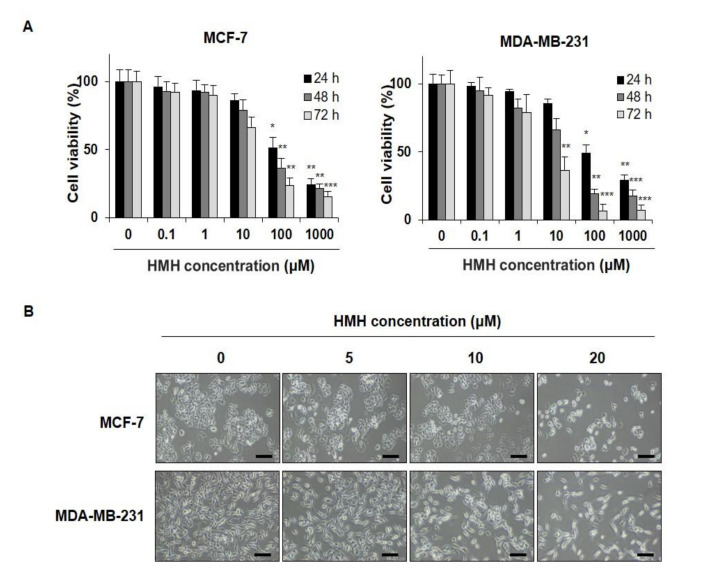
Effects of harmine hydrochloride on the growth of human breast cancer cell lines—MCF-7 and MDA-MB-231. (**A**) Cell survival curve in breast cancer cell lines following harmine hydrochloride treatment. The cells were cultured with 0, 1, 10, 100, and 1000 µM harmine hydrochloride for 24, 48, and 72 h, followed by measurement of cell proliferation through MTT analysis. (**B**) Morphological changes. Harmine hydrochloride suppressed the growth of MCF-7 and MDA-MB-231 cells in a dose-dependent manner to reduce cell density. Results are expressed as mean ± SD. Statistical differences were analyzed using Student’s *t*-test (* *p* < 0.05, ** *p* < 0.01, *** *p* < 0.001 vs. control). Cell morphology was visualized by inverted microscopy (200×). Scale bar, 50 µm.

To determine whether HMH was effective in suppressing cell proliferation in human BC cells, MCF-7 and MDA-MB-231 cells were treated with HMH at various concentrations, ranging from 0 to 1000 μM for 24–72 h, and cell proliferation was measured. After HMH treatment for 24, 48, and 72 h, the IC_50_ value was found to be 100.6, 52.4, and 18.7 μM in MCF-7 cells and 91.9, 17.7, and 6.1 μM in MDA-MB-231 cells, respectively (Figure 2A). In addition, significant suppression of cell proliferation was identified for all the lengths of treatment with ≥100 μM of HMH (*p* < 0.05, *p* < 0.01, *p* < 0.001) following the identification of the biologically active range of HMH concentrations based on the IC_50_ and morphological changes. In Figure 2B, inhibition of cell proliferation can be observed after HMH treatment, and after 20 μM HMH treatment, MCF-7 cells exhibited shrinkage in size compared to control. Moreover, MDA-MB-231 cells became more elongated. MCF-7 and MDA-MB-231 cells were treated with 0–20 μM of HMH for further experiments.

### 2.2. HMH Inhibits the Migration of BC Cells

**Figure 3:**
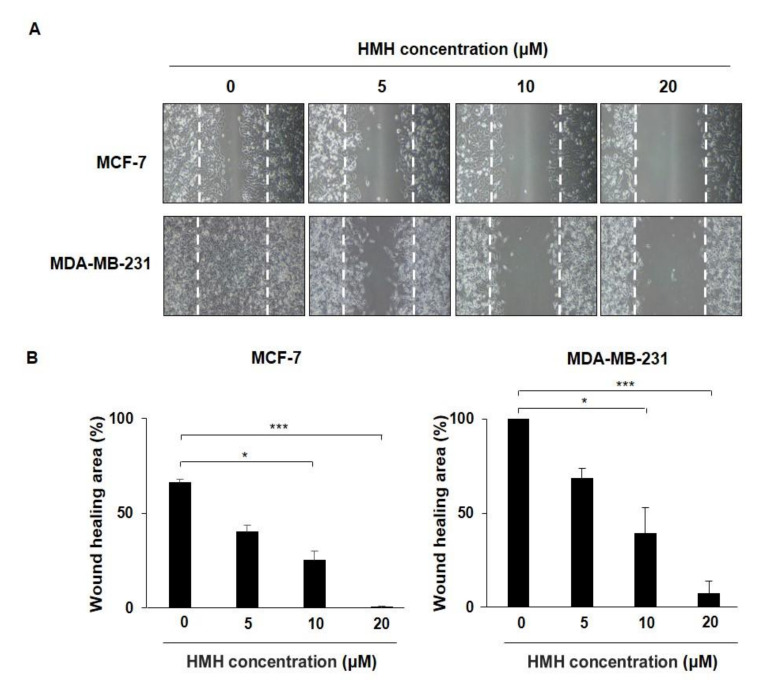
Effects of harmine hydrochloride on the migration of MCF-7 and MDA-MB-231 cells. (**A**) Upon confluency in six-well plates, the MCF-7 and MDA-MB-231 cells were scratch-wounded and treated with harmine hydrochloride at various concentrations in the range of 0–20 μM to measure the migration area after 48 h. (**B**) The MCF-7 and MDA-MB-231 cells were observed with a microscope, followed by measurement of the wound area, and the percentage of the healed area has been represented after quantification using ImageJ software. Values are presented as mean ± SD. * *p* < 0.05, *** *p* < 0.001, as shown by the Student’s *t*-test.

For migration analysis, the BC cells at a confluency of 70–80% were scratched with a P20 pipette tip, and the amount of FBS added to the cell culture media was reduced from 10% to 1%. HMH was then applied at concentrations of 0–20 μM for 48 h to observe migration. As shown in Figure 3A, the wound healing area of the MCF-7 cells was 66.2% in the control group and 0.2% in the HMH 20 μM treatment group, showing that migration was significantly inhibited (*p* < 0.001). In addition, the wound healing area of the MDA-MB-231 cells after 48 h was 100% in the control group but 7.4% in the HMH 20 μM treatment group, also showing that the migration was significantly inhibited (*p* < 0.001). This has been measured and plotted in Figure 3B. These results indicate that HMH can significantly inhibit the migration of both the BC cell lines being investigated (*p* < 0.001).

### 2.3. HMH Inhibits the Invasion of BC Cells

**Figure 4:**
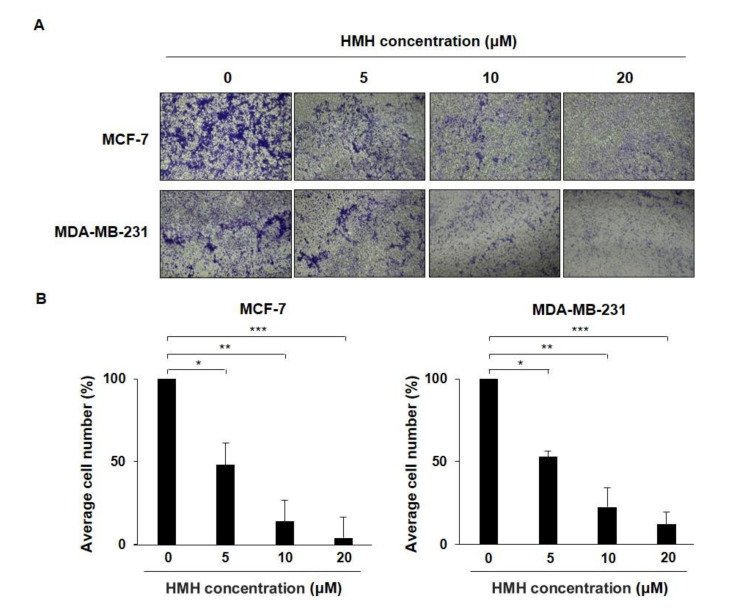
Effects of harmine hydrochloride on the invasion of MCF-7 and MDA-MB-231 cells. (**A**) MCF-7 and MDA-MB-231 cells were separately placed into the upper chamber with 1.5 mL of serum-free Dulbecco’s modified Eagle medium (DMEM), and DMEM containing 10% FBS was added to the lower chamber. After 48 h of culturing, the non-invading cells in the upper chamber were removed with a swab. Cells invading the lower chamber were fixed with 4% paraformaldehyde, stained with 0.5% crystal violet solution, and observed under a microscope. (**B**) Dyed MCF-7 and MDA-MB-231 cells were observed under a microscope, and the invading cells were measured and quantified using ImageJ software. Values are presented as mean ± SD. * *p* < 0.05, ** *p* < 0.01, *** *p* < 0.001, as shown by the Student’s *t*-test.

To identify the degree of BC cell invasion, HMH was applied for 48 h at 0–20 μM with BC cells in a chamber containing 8 µm polycarbonate membranes and the number of cells that moved to the lower chamber was measured compared to that of the control group (Figure 4A). As shown in Figure 4B, the number of invading cells in the 20 μM HMH treatment group compared to that in the control group was 3.98% for the MCF-7 cells and 12.14% for the MDA-MB-231 cells, which were both significantly reduced (*p* < 0.001).

### 2.4. HMH Inhibits Colony Formation of BC Cells

**Figure 5:**
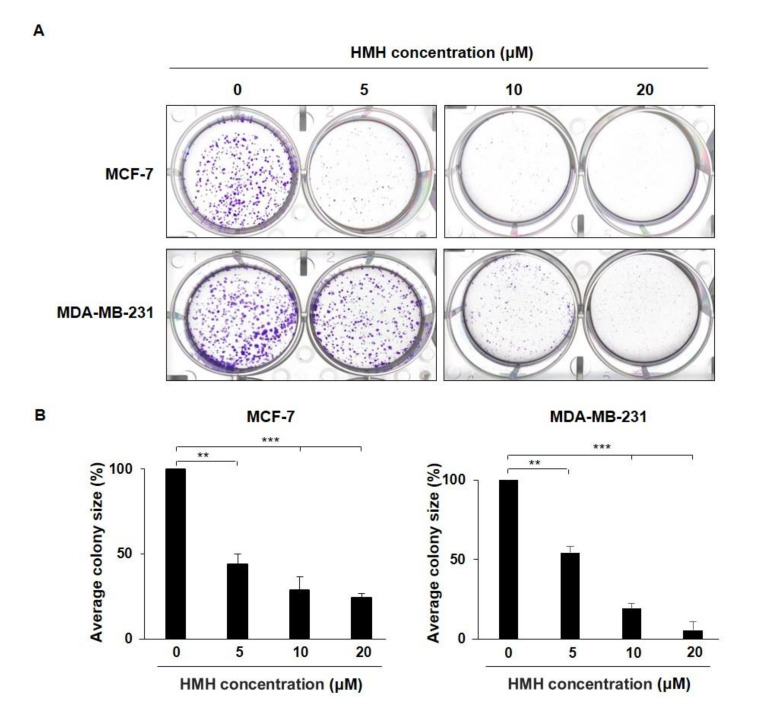
Effects of harmine hydrochloride on colony formation by MCF-7 and MDA-MB-231 cells. (**A**) The MCF-7 and MDA-MB-231 cells were each seeded into six-well plates followed by treatment with harmine hydrochloride at various concentrations of 0–20 μM for 24 h and removal of the media. Thereafter, the cells were cultured for 14 days along with the replacement of media with fresh media every 3–4 days. After identification of colony formation, they were fixed with methanol and dyed with 0.5% crystal violet. (**B**) After the observation of the MCF-7 and MDA-MB-231 cells with a microscope, colony size was measured, and the cells were quantified using ImageJ software. Values are presented as means ± SD. ** *p* < 0.01, *** *p* < 0.001, as shown by the Student’s *t*-test.

To evaluate the suppressive effects of HMH on colony formation, the BC cells were treated with HMH at various concentrations for 24 h. Then, the medium containing HMH was removed, and the cells were cultured in fresh media for 14 days to identify colony formation (Figure 5A). As shown in Figure 5B, the size of colony formation in the HMH 20 μM treatment group compared to that of the control was 24.15% for the MCF-7 cells and 5.51% for the MDA-MB-231 cells, showing a significant reduction (*p* < 0.001).

### 2.5. HMH Induces G2/M Cell Cycle Arrest in BC Cells

**Figure 6:**
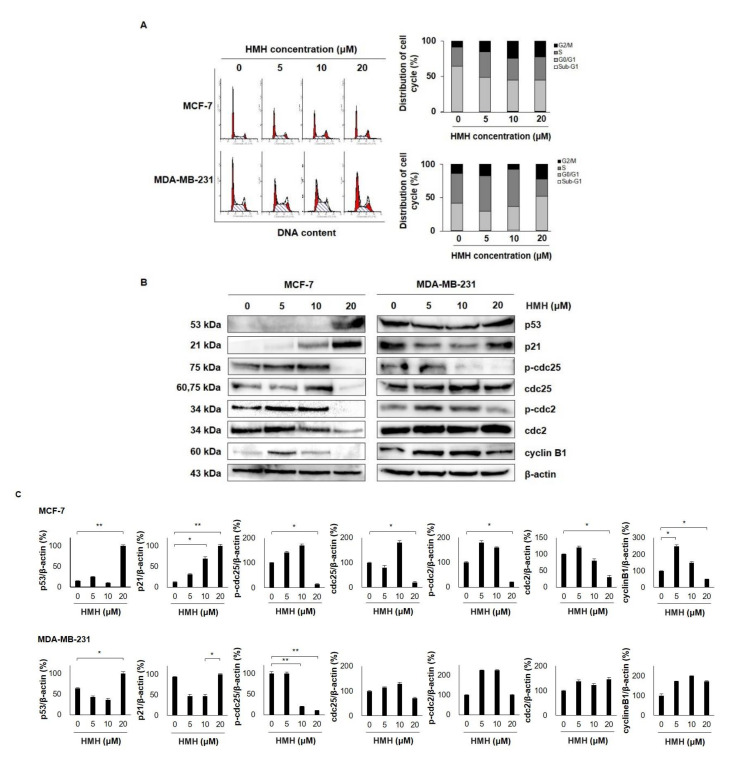
Effects of harmine hydrochloride on the cell cycle progression of MCF-7 and MDA-MB-231 cells. (**A**) The MCF-7 and MDA-MB-231 cells were treated with harmine hydrochloride (0, 5, 10, and 20 µM) for 48 h. Then, the cells dyed with propidium iodide (PI) were analyzed with flow cytometry to identify cell cycle progression. The cumulative distribution of the cells in Sub-G1, G0/G1, S, and G2/M phases was identified in the MCF-7 and MDA-MB-231 cells following harmine hydrochloride treatment. Harmine hydrochloride treatment induced the G2/M cell cycle in a dose-dependent manner. (**B**) The expression levels of proteins associated with G2/M, including p53, p21, p-cdc2, cdc2, and cyclin B1, were identified in MCF-7 and MDA-MB-231 cells using western blot. β-actin was used as an internal control. Full images can be found in the Supplemental Materials. (**C**) Band intensity was normalized using β-actin. Values indicate the mean ± SD. * *p* < 0.05, ** *p* < 0.01 as shown by the Student’s *t*-test.

As we determined the suppressive effects of HMH on cell proliferation, additional studies on the cell cycle regulation of MCF-7 and MDA-MB-231 cells by HMH were conducted. Treatment with 0–20 μM HMH for 48 h revealed that the fraction of cells in the G2/M phase in the control group was 8.69% and 13.76% for MCF-7 and MDA-MB-231 cells, respectively, which increased to 22.50% and 22.64%, respectively, in the 20 μM HMH group (Figure 6A). Following this, the cell cycle regulatory proteins associated with the G2/M phase were identified through western blot analysis. As shown in Figure 6B, the expression of p53 and p21, the cell cycle regulatory factors, increased in MCF-7 and MDA-MB-231 cells following HMH treatment. In addition, HMH dose-dependently decreased the expression of p-cdc25, cdc25, p-cdc2, cdc2, and cyclin B1, the G2/M phase regulatory proteins, in MCF-7 cells. Similarly, its dose-dependence reduced the expression of p-cdc25, p-cdc2, and cyclin B1, the G2/M phase regulatory proteins, in MDA-MB-231 cells, whereas the expression of cdc25 and cdc2 did not vary with dose. In particular, the expression of p-cdc25, cdc25, p-cdc2, cdc2, and cyclin B1 in MCF-7 cells was not statistically different between 5 and 10 μM HMH and control groups. However, it was significantly decreased in the 20 μM HMH treatment group (*p* < 0.05), and the expression of p-cdc25 in MDA-MB-231 cells was significantly reduced in the 10 and 20 μM HMH treatment group (*p* < 0.01) compared to that in the control group (Figure 6C).

### 2.6. HMH Regulates the MAPKs and AKT/FOXO3a Signaling Pathways in BC Cells

**Figure 7:**
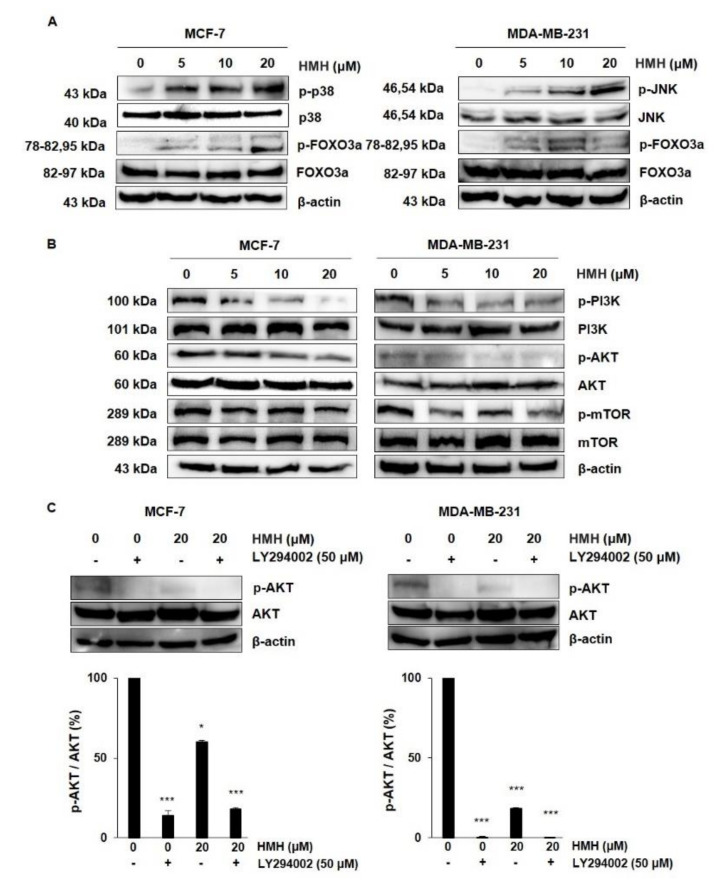
Action of harmine hydrochloride on MAPK and PI3K/AKT/mTOR signaling pathways in MCF-7 and MDA-MB-231 cells. (**A**) MAPK-associated protein expressions following treatment with harmine hydrochloride at 5, 10, and 20 μM for 48 h in the MCF-7 and MDA-MB-231 cells are shown in the representative blots. (**B**) PI3K/AKT/mTOR signal-associated protein expressions following treatment with harmine hydrochloride at 5, 10, and 20 μM for 48 h in MCF-7 and MDA-MB-231 cells are shown as representative blots. β-actin was used as an internal control. (**C**) Additionally, 0 or 20 μM of harmine hydrochloride was applied in combination with the AKT inhibitor LY294002 to the MCF-7 and MDA-MB-231 cells to identify the p-AKT expression through western blot. Band intensity was normalized using the total AKT. Values indicate the mean ± SD. * *p* < 0.05 and *** *p* < 0.001, as shown by the Student’s *t*-test.

P38 and JNK are the most typical kinases among MAPKs that link important extracellular signals that regulate cell proliferation, differentiation, migration, and apoptosis. The expression of MAPKs was different between MCF-7 and MDA-MB-231 cells. As shown in Figure 7A, HMH treatment dose-dependently increased the expression of p-p38 and p-FOXO3a in MCF-7 cells, whereas in MDA-MB-231 cells, the levels of p-JNK and p-FOXO3a were higher. The PI3K/AKT/mTOR signaling pathway is also a good regulator of cell proliferation and the metastatic process, allowing us to identify whether the pathway was associated with HMH treatment of BC cells (Figure 7B). To evaluate the suppressive effects on protein expression following HMH treatment, western blot analysis was conducted for BC cells treated with 5, 10, and 20 μM HMH for 48 h. In both MCF-7 and MDA-MB-231 cells, HMH dramatically decreased the expression of p-PI3K and p-AKT in a dose-dependent manner, and p-mTOR expression decreased by 20 μM HMH compared to that in the control. Therefore, the cells were treated with LY294002, an AKT inhibitor, in combination with 0 or 20 μM HMH to determine whether HMH could function as a PI3K/AKT inhibitor (Figure 7C). Quantification of the western blots indicate that HMH significantly inhibited p-AKT activation in both MCF-7 and MDA-MB-231 cells (*p* < 0.05, *p* < 0.001).

## 3. Discussion

Although current strategies for BC treatment, including hormone therapy and chemotherapy, can lead to substantial anticancer activity, the development of new anticancer drugs derived from natural products with low toxicity remains an area of interest. Harmine is a natural β-carboline alkaloid isolated from *Peganum harmala*, which was been previously used in folk medicine as an anticancer therapy [32]. Studies have shown that harmine exhibits significant antitumor activities, including inhibiting proliferation, migration, and invasion [8,33,34], and inhibits the growth of several types of cancers, including lung [35], gastric [36], and hepatic cancer [37]. HMH has identical pharmacological activities to harmine, including anti-Alzheimer’s disease, anticancer, and anti-inflammatory activities [38,39]. However, the detailed molecular mechanisms underlying its activity in BC cells are currently unknown.

ER-positive MCF-7 cells and TNBC MDA-MB-231 cells are commonly used in comparative studies of BC cells related to receptor heterogeneity [40,41]. In this study, HMH suppressed the growth of ER-positive MCF-7 cells, TNBC MDA-MB-231 cells, and their proliferation by inducing G2/M arrest. These results imply that the suppressive effects of HMH on the growth of BC cells are independent of hormone signaling. Following the suppression of BC cell proliferation by HMH, its inhibitory effects on migration, invasion, and colony formation were identified. In addition to MCF-7 cells, HMH significantly reduced the migration and colony formation of aggressive human BC MDA-MB-231 cells. Therefore, HMH could be a highly potent active compound responsible for anticancer bioactivity.

The PI3K/AKT/mTOR signaling pathway is an important intracellular signaling pathway essential for cell proliferation, metabolism, and angiogenesis that functions by affecting the activities of downstream molecules. It is also closely associated with the development and progression of human tumors. AKT signaling regulates cell proliferation [42], and FOXO3a is an important target of the PI3K/AKT signaling pathway [43] that regulates cell cycle arrest by activating transcriptional targets, such as p27 and p21 [44]. The results of this study show that HMH inhibits AKT phosphorylation in MCF-7 cells and increases FOXO3a phosphorylation in a dose-dependent manner. Similarly, inhibition of AKT phosphorylation and increase in FOXO3a phosphorylation compared to the control following HMH treatment were identified in MDA-MB-231 cells. JNK and p38 activate FOXO3a in response to oxidative stress. Our results also showed that HMH treatment dose-dependently increased p38 phosphorylation in MCF-7 cells and JNK phosphorylation in MDA-MB-231 cells to activate FOXO3a.

FOXO3a promotes the expression of target genes, such as p21, p27, p53, cyclin D, and cyclin B, causing cell cycle arrest that inhibits cancer cell growth [45]. The cell cycle is a common phenomenon in eukaryotic cell division, and cell cycle progression includes four main checkpoints: G1/S, S, G2/M, and spindle assembly [46]. Cyclins and CDK complexes regulate cell cycle progression, with differential activity in different cell cycle stages. The G1 and S phases are regulated by CDK2, CDK6, CDK4, cyclin D1, and cyclin E, whereas the G2/M phase is controlled by CDK2, cdc2, cyclin A, and cyclin B. In eukaryotic cells, the cyclin B/cdc2 complex plays an important role in controlling the G2/M transition. p21^Waf1/Cip1^, a CDK inhibitor, inactivates the cyclin B1/cdc2 complex in p53-dependent sustained G2/M arrest [47]. In this study, we identified the induction of G2/M cell cycle arrest by HMH in both MCF-7 and MDA-MB-231 BC cells. Immunoblotting results showed that HMH remarkably upregulated p53 and p21 and downregulated p-cdc25, p-cdc2, and cyclin B1 in BC cells at the protein level.

## 4. Materials and Methods

### 4.1. Materials

HMH (Figure 1) was purchased from Sigma-Aldrich (St. Louis, MO, USA) and dissolved in 100% dimethyl sulfoxide (DMSO). A 50 mM stock solution of HMH was prepared and stored as small aliquots at −20 °C until needed. DMSO, 3-(4,5-Dimethylthiazol-2-yl)-2,5-diphenyltetrazolium bromide (MTT), and horseradish peroxidase (HRP)-conjugated anti-rabbit and anti-mouse antibodies were purchased from Sigma-Aldrich. Phospho-specific anti-p38, anti-ERK, anti-FoxO3a, anti-cdc25, anti-PI3K, anti-AKT, and anti-mTOR, and specific antibodies anti-p38, anti-ERK, anti-FoxO3a, anti-cdc25, anti-PI3K, anti-AKT, anti-mTOR, and AKT inhibitor LY294002 were purchased from Cell Signaling Technology (Danvers, MA, USA). HRP-conjugated β-actin, p53, p21, p-cdc2, cdc2, and cyclin B1 antibodies were purchased from Santa Cruz Biotechnology (Santa Cruz, CA, USA).

### 4.2. Cell Culture

MCF-7 and MDA-MB-231 human breast cancer cell lines were purchased from the American Type Culture Collection (ATCC; Rockville, MD, USA). The cells were grown using a 5% CO_2_ incubator at 37 °C in Dulbecco’s modified Eagle medium (DMEM) containing 10% fetal bovine serum (FBS) and 1% antibiotic–antimycotic agent.

### 4.3. MTT Assay

The survival rates of MCF-7 and MDA-MB-231 cells following HMH treatment were measured using the MTT assay. The MCF-7 and MDA-MB-231 cells were seeded in separate 96-well plates and cultured overnight, followed by removal of the culture medium, addition of DMEM treated with 0–1000 μM HMH, and culturing for 24–72 h. Next, 20 μL of MTT was added to each well at a concentration of 5 mg/mL and cultured for 3–4 h. Then, the medium was removed, and 200 μL of 100% DMSO was added to each well to stop the reaction and dissolve the formazan. The Synergy HTX plate reader (Bio-Tek Instruments, Inc., Winooski, VT, USA) and Gen5 software were used to measure the OD value at 570 nm and cell viability, respectively.

### 4.4. Wound Healing Assay for the Migration Assay

The cells were seeded in six-well plates and cultured for 24 h. A P20 pipette tip was used to create an artificial wound in the cells in each well. Dead cells due to the wound were washed with fresh medium, followed by the addition of 0–20 µM HMH to fresh medium containing 1% FBS for 24–72 h. Following culture for 24–72 h, an inverted microscope equipped with a camera was used to obtain images. The wound healing area was quantified using ImageJ software.

### 4.5. Invasion Assay

The cell invasion assay was conducted using six-well dishes that included Transwell inserts made of 8 µm polycarbonate membranes (Corning Incorporated Costar, Tewksbury, MA, USA). Briefly, 1.5 mL of serum-free DMEM with cells was added to the upper chamber (BD BioCoat^TM^ Matrigel^TM^ Invasion Chamber; BD Biosciences, San Diego, CA, USA), while DMEM containing 10% FBS was added to the lower chamber. After incubation in a 5% CO_2_ incubator at 37 °C for 48 h, non-invading cells in the upper chamber were removed with a swab for analysis of both the control and HMH treatment groups. The cells invading the lower chamber were fixed in 4% paraformaldehyde for 30 min and then dyed with 0.5% crystal violet solution for 30 min. Following staining, the invasive cells were observed and quantified under a microscope.

### 4.6. Colony Formation Assay

In total, 1000 cells/well were added to 2 mL of DMEM containing 10% FBS and seeded in six-well plates. The cells were cultured for 24 h in media treated with various concentrations of HMH, followed by removal of the media. They were then replaced with fresh media without HMH every 3–4 days at 37 °C and grown in a 5% CO_2_ incubator for 14 days to observe colony formation. Cell colonies were fixed in methanol for 15 min and stained with 0.1% crystal violet for 10 min. The colonies (>50 cells) were counted under a microscope (Olympus FV500; Olympus Corporation, Tokyo, Japan).

### 4.7. Flow Cytometry Analysis for Cell Cycle Distribution

The cells were spread on a 100 mm culture dish and incubated for 24 h. When the cell density was >70%, 0–20 μM HMH-containing culture medium was added for 48 h. The cells were then harvested using trypsin/EDTA and fixed overnight in cold 70% ethanol at −20 °C. For cell cycle analysis, the fixed cells were centrifuged for 5 min at 5000 rpm and 4 °C, washed with cold PBS, and treated with 50 µg/mL RNase A at 37 °C for 30 min. The cells were stained with 50 µg/mL propidium iodide (PI) at 37 °C for 30 min. The DNA content of the stained cells was analyzed using a FACS Vantage SE flow cytometer and the CellQuest software (BD Biosciences, San Diego, CA, USA).

### 4.8. Determination of Protein Expression by Western Blotting

The cells were treated with HMH (0–20 μM) for 48 h. Next, the PRO-PREP protein extraction solution containing protease inhibitors and phosphatase inhibitors (Roche Diagnostics GmbH, Mannheim, Germany) was used to extract proteins at 4 °C for 30 min, followed by centrifugation for 30 min at 13,000 rpm and 4 °C. The protein samples (30–50 µg) were separated by 6–12% SDS-PAGE and transferred to polyvinylidene fluoride membranes (Bio-Rad Laboratories, Inc., Hercules, CA, USA). The membranes were blocked for 1 h using Tris-buffered saline (TBS-T) with 5% BSA (AMRESCO, Cleveland, OH, USA) and 0.1% Tween 20. Then, they were incubated overnight at 4 °C with 5% BSA diluted with primary antibodies (1:500~1:1000). Next, the membranes were washed four times (3 min each) with TBS-T. After washing, the membranes were incubated with HRP-conjugated anti-rabbit or anti-mouse secondary antibodies (1:1000) for 1 h at room temperature, followed by detection using an Advanced Electrochemiluminescence Western Blot Detection Kit (Amersham, Uppsala, Sweden).

### 4.9. Statistical Analysis

Data are presented as mean ± SD for the indicated number of independent experiments. Statistical significance (*p* < 0.05) was determined using the Student’s *t*-test for paired data. Statistical analyses were performed using SPSS for Windows (v23.0; SPSS, Chicago, IL, USA).

## 5. Conclusions

In this study, HMH treatment of BC cells inactivated the PI3K/AKT/mTOR pathway and increased the expression of FOXO3a. In addition, it activated p38 phosphorylation in MCF-7 cells and JNK phosphorylation in MDA-MB-231 cells. Inactivation of the PI3K/AKT/mTOR pathway and activation of p38 and JNK provoked FOXO3a expression, which regulated the expression of p53, p21, and cyclin B1 to induce G2/M cell cycle arrest. Moreover, HMH functioned as an inhibitor of the PI3K/AKT/mTOR pathway by decreasing p-AKT expression in the group treated with a combination of AKT inhibitors. Thus, these results show that MAPKs and AKT/FOXO3a signaling pathways mediate the induction of cell cycle arrest following HMH treatment, implying that HMH could be a potential active compound responsible for anticancer bioactivity towards BC.

## Data Availability

All data generated or analyzed during this study are included in the published article.

## Supplementary Materials

The following are available online, Western blot images of Figure 6 and Figure 7.

## Sample Availability

Samples of the compounds are not available from the authors.
