# Supplementary material for: Harmine Hydrochloride Mediates the Induction of G2/M Cell Cycle Arrest in Breast Cancer Cells by Regulating the MAPKs and AKT/FOXO3a Signaling Pathways
Source: Molecules. 2021 Nov 5;26(21):6714. doi: 10.3390/molecules26216714 (PMC8588485; doi:10.3390/molecules26216714)
Supplement: Supplementary file 1 [file molecules-26-06714-s001.zip › molecules-1395026-supplementary.pdf]

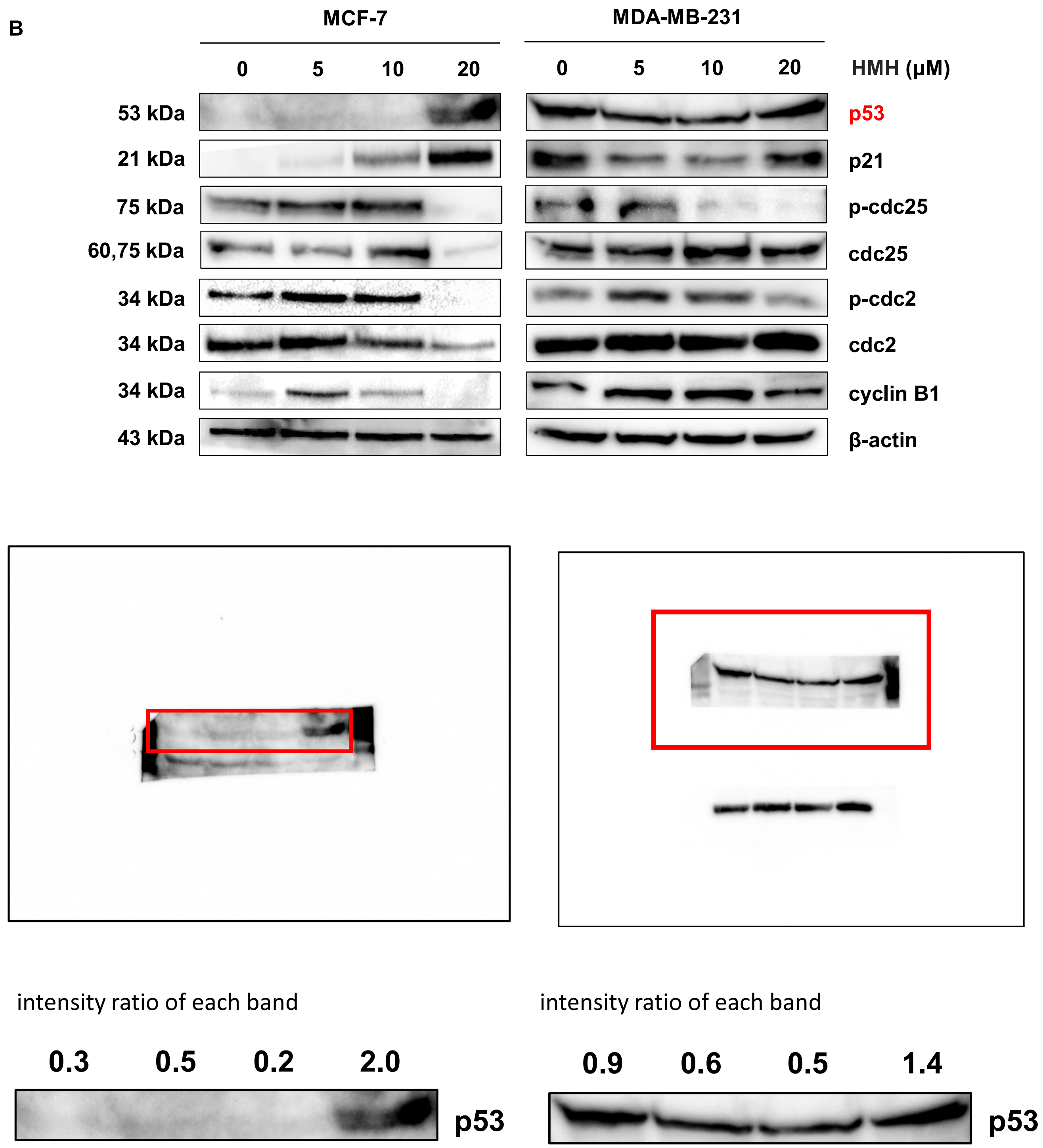
B
MCF-7
MDA-MB-231
0
5
10
20
0
5
10
20
HMH (μM)
53 kDa
21 kDa
75 kDa
60,75 kDa
34 kDa
34 kDa
34 kDa
43 kDa
p53
p21
p-cdc25
cdc25
p-cdc2
cdc2
cyclin B1
β-actin
intensity ratio of each band
0.3
0.5
0.2
2.0
p53
intensity ratio of each band
0.9
0.6
0.5
1.4
p53

F6

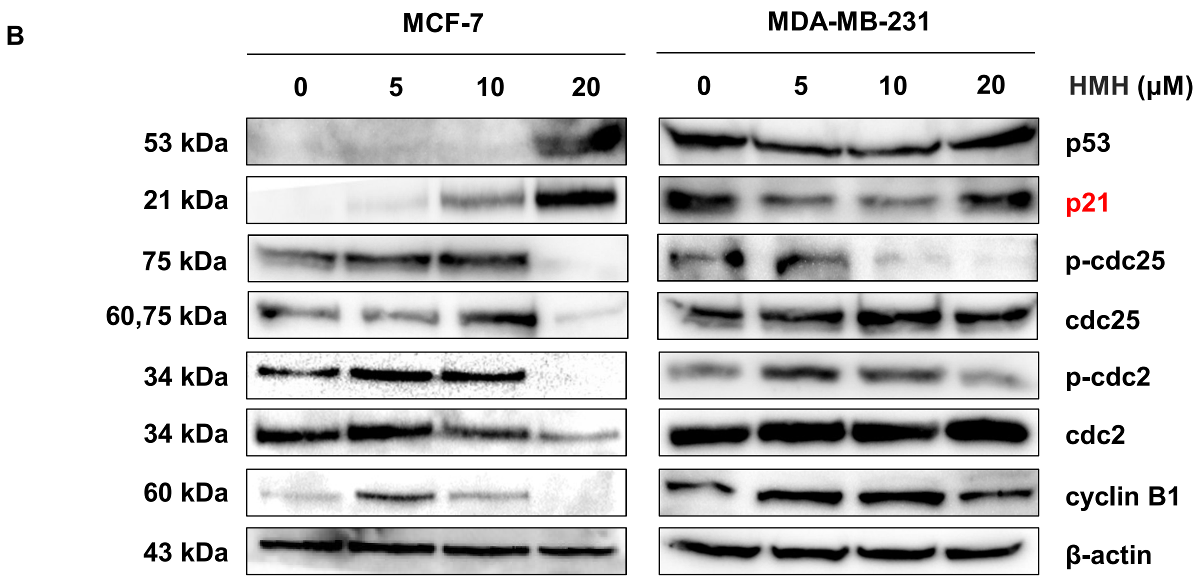

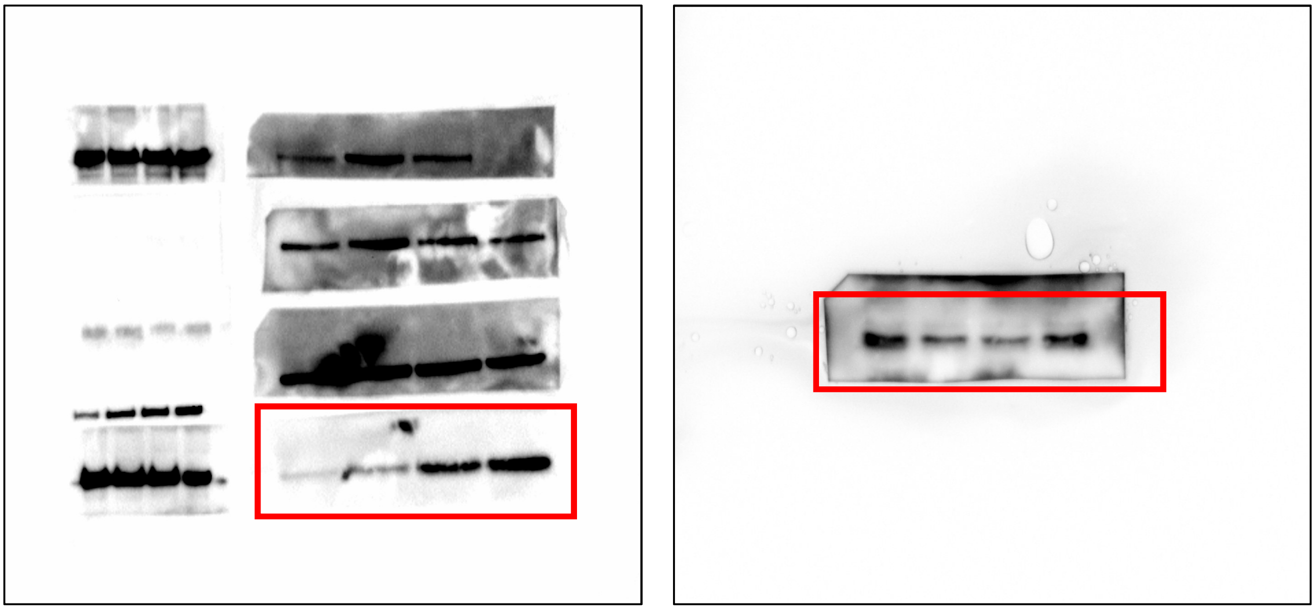

intensity ratio of each band

intensity ratio of each band

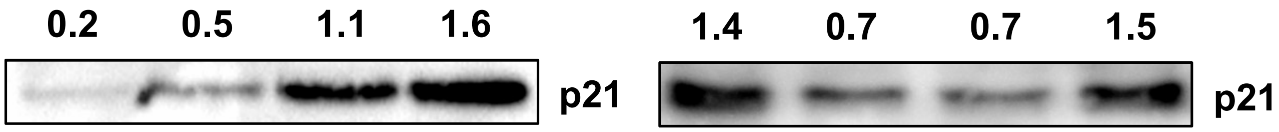

F6

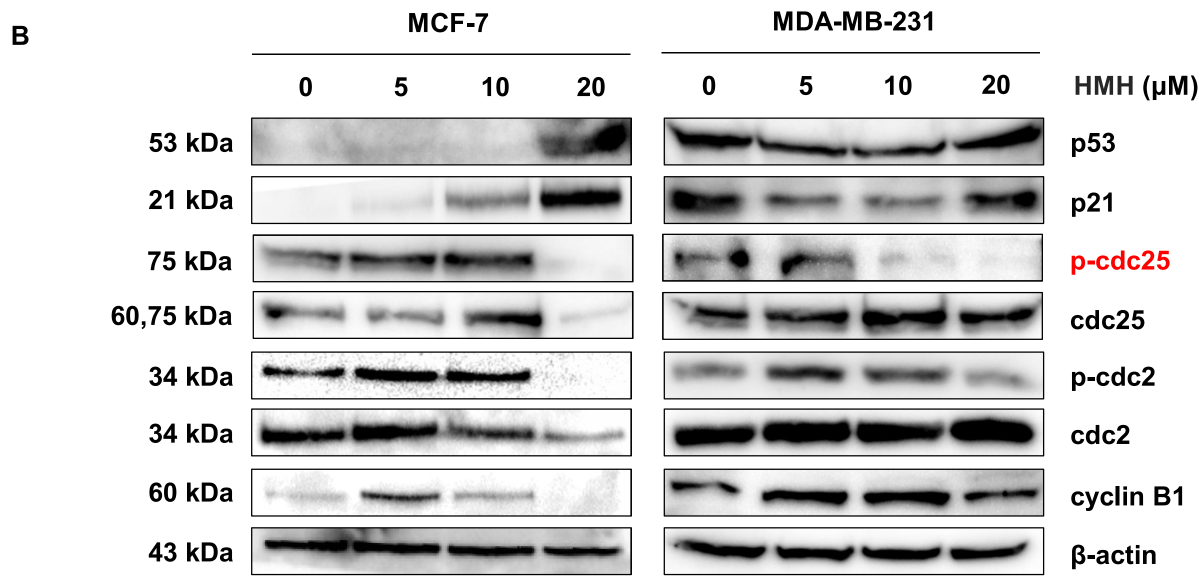

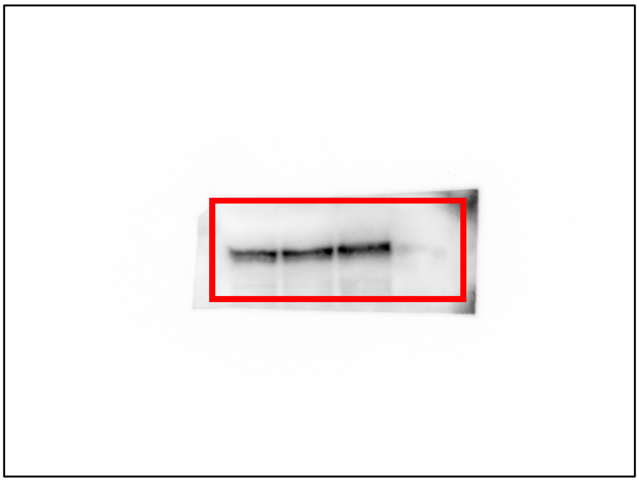

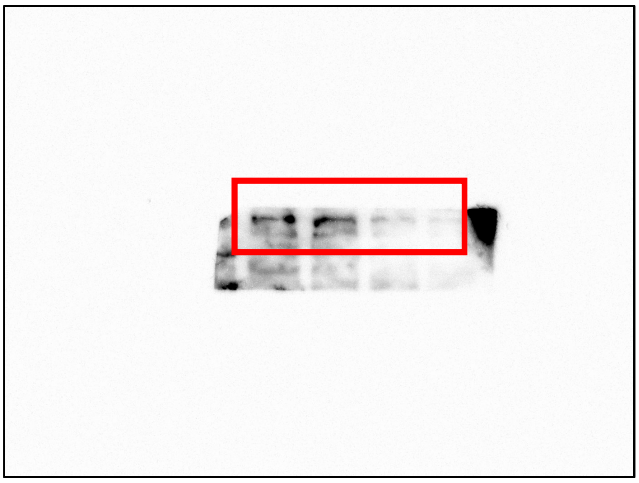

intensity ratio of each band

0.7 1.0 1.2 0.1

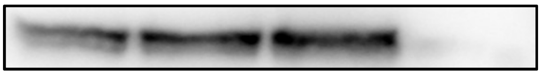

p-cdc25

intensity ratio of each band

1.0 1.0 0.2 0.1

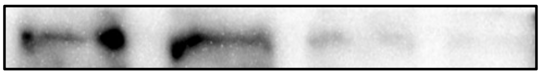

p-cdc25

F6

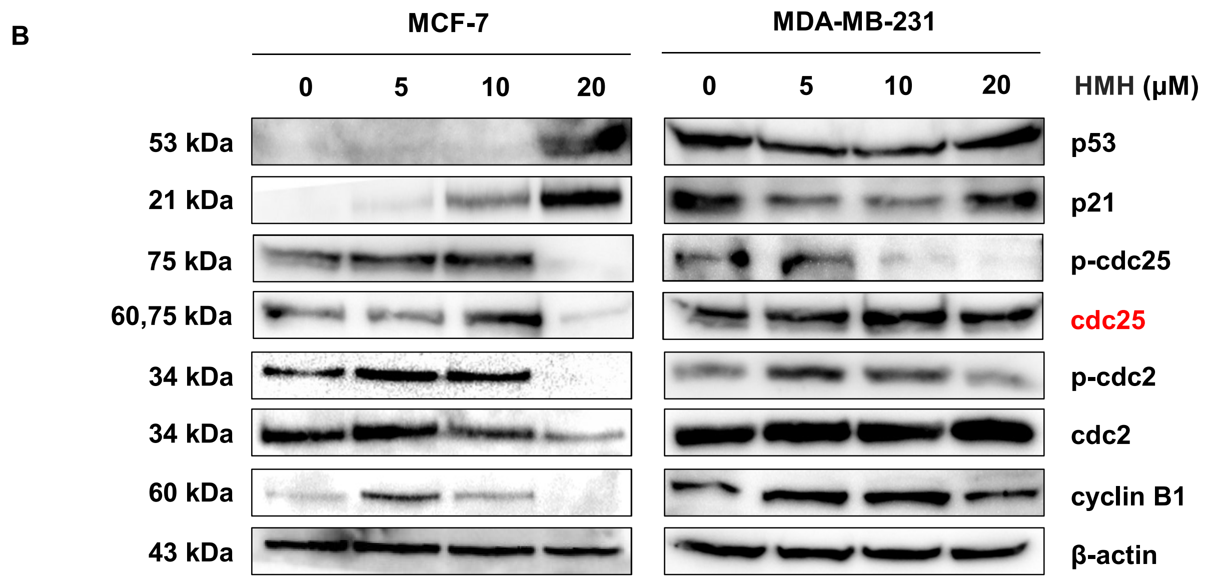

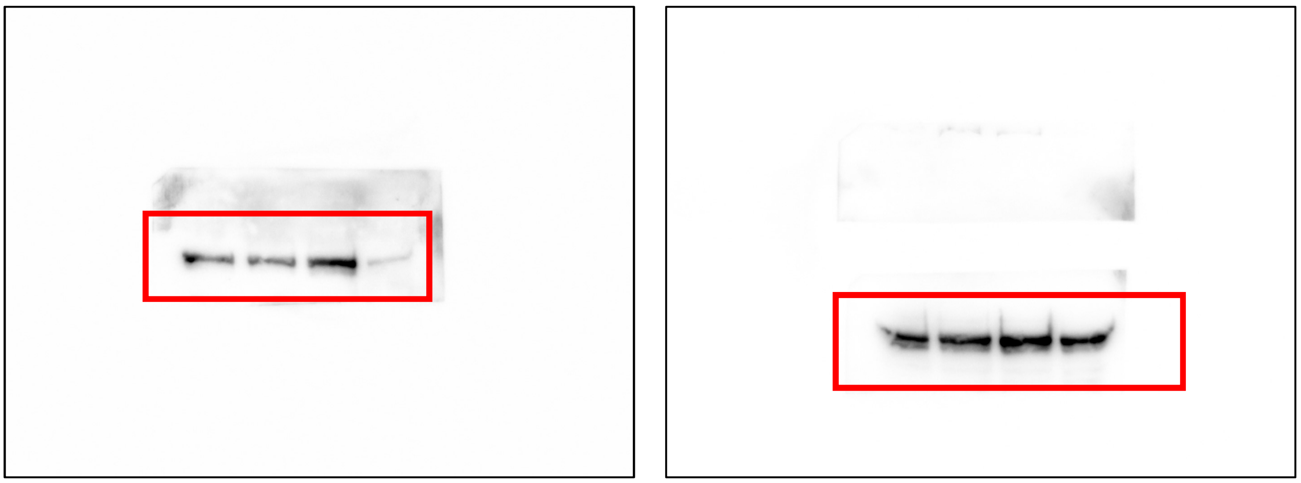

intensity ratio of each band

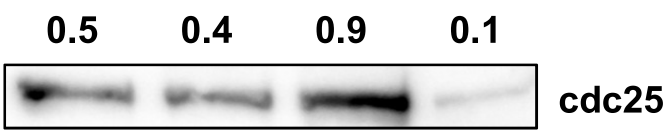

intensity ratio of each band

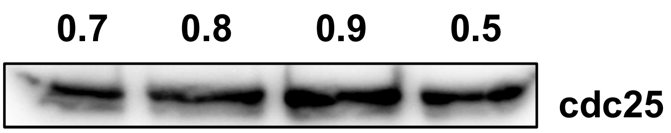

F6

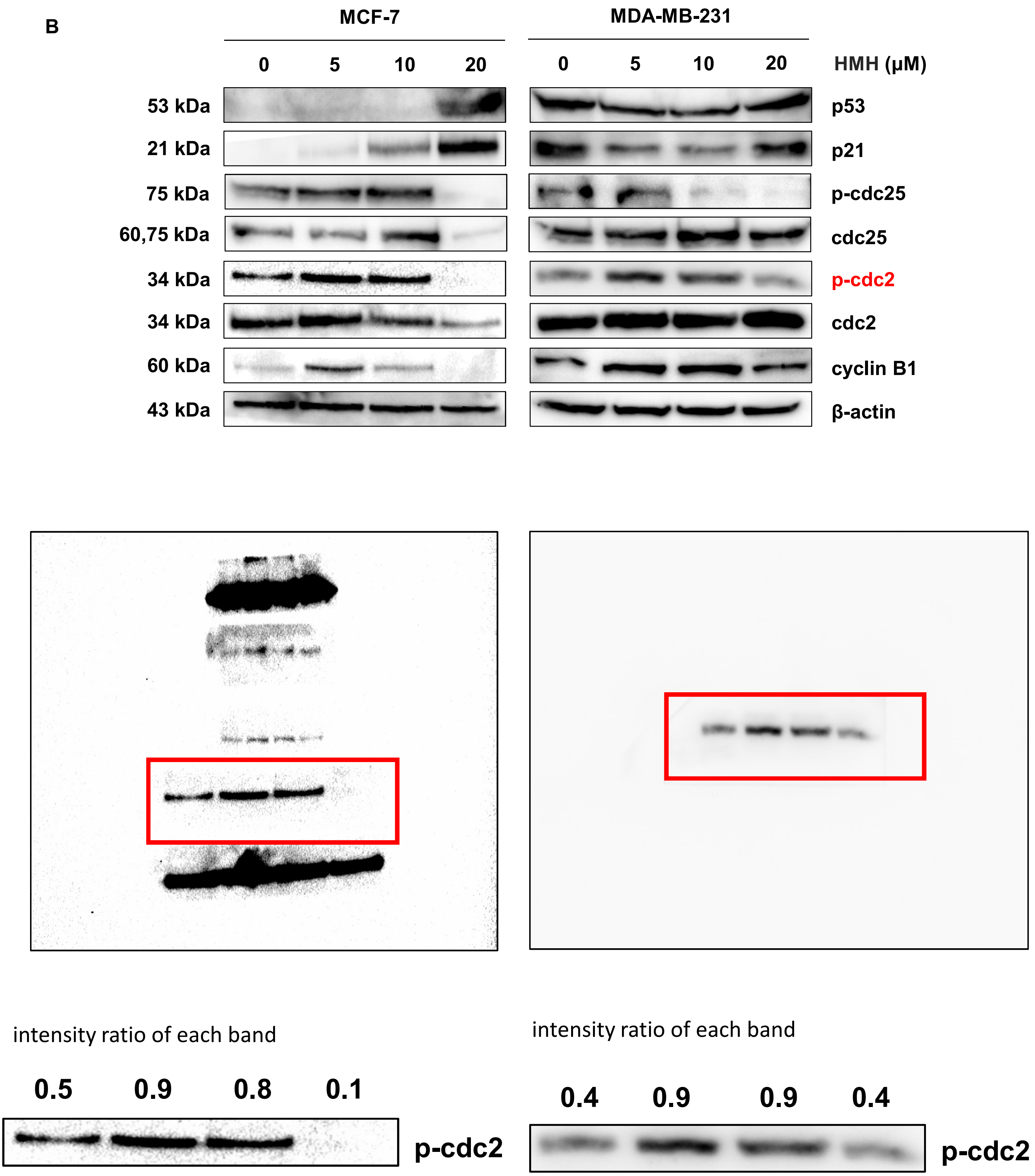
B
MCF-7
MDA-MB-231
0
5
10
20
HMH (μM)
53 kDa
21 kDa
75 kDa
60,75 kDa
34 kDa
34 kDa
60 kDa
43 kDa
p53
p21
p-cdc25
cdc25
p-cdc2
cdc2
cyclin B1
β-actin
intensity ratio of each band
0.5 0.9 0.8 0.1
p-cdc2
intensity ratio of each band
0.4 0.9 0.9 0.4
p-cdc2

F6

B

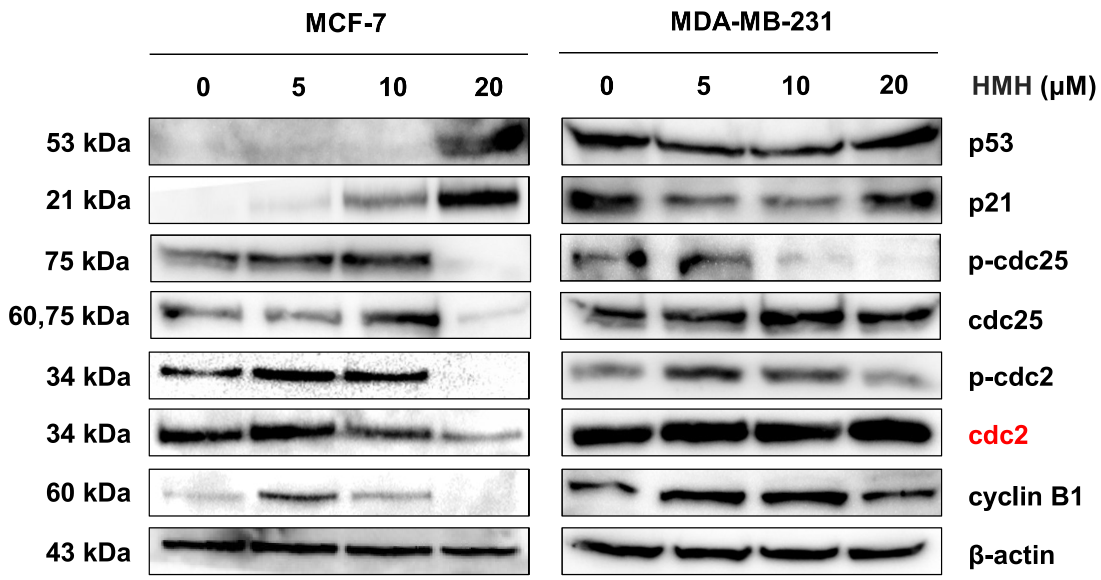

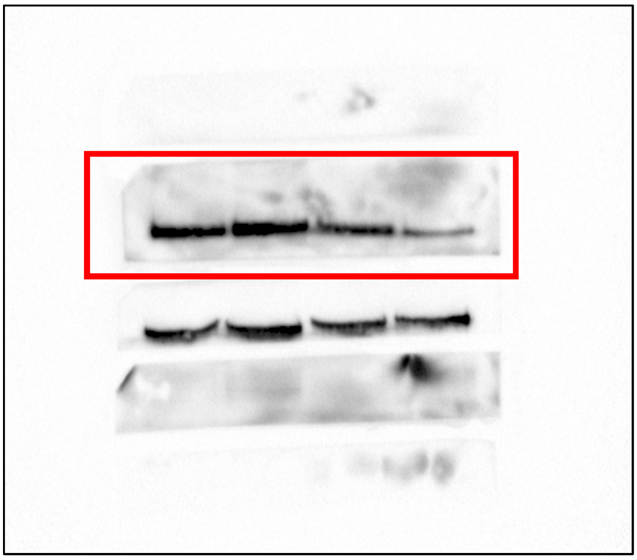

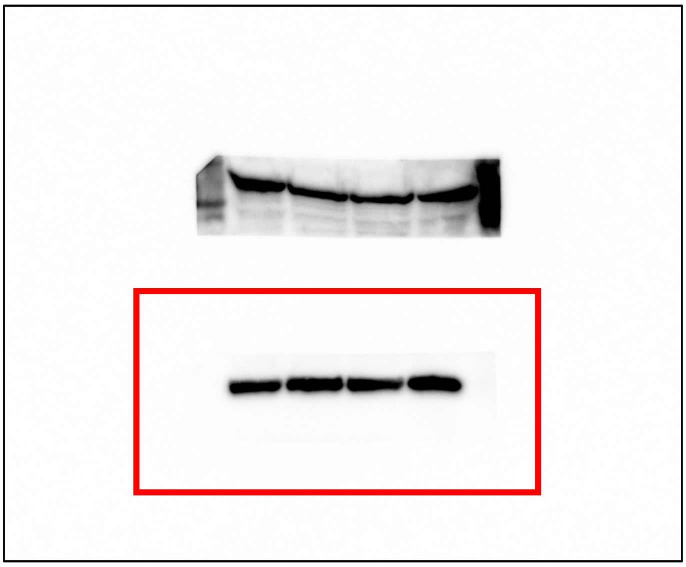

intensity ratio of each band

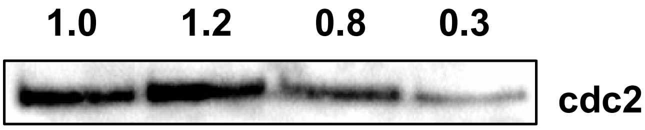

intensity ratio of each band

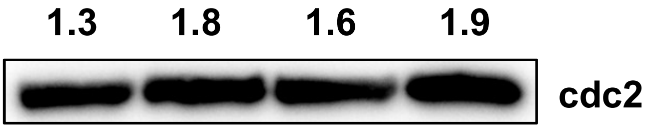

F6

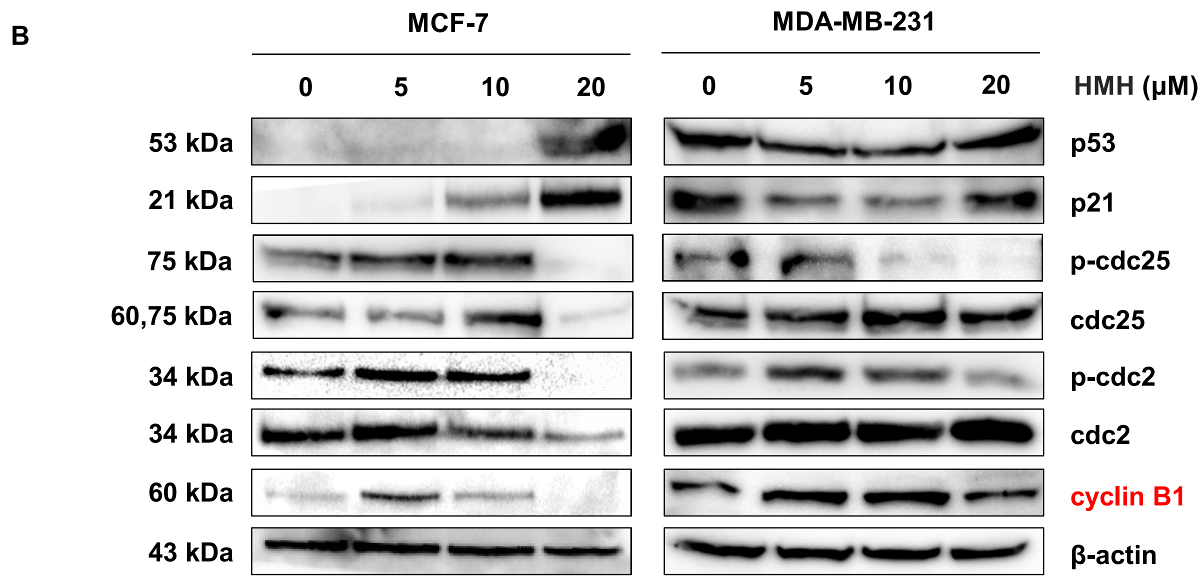

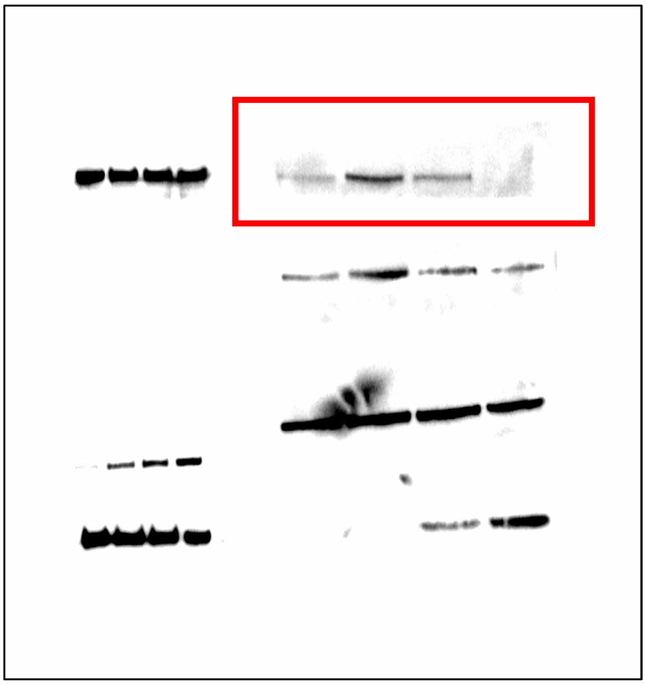

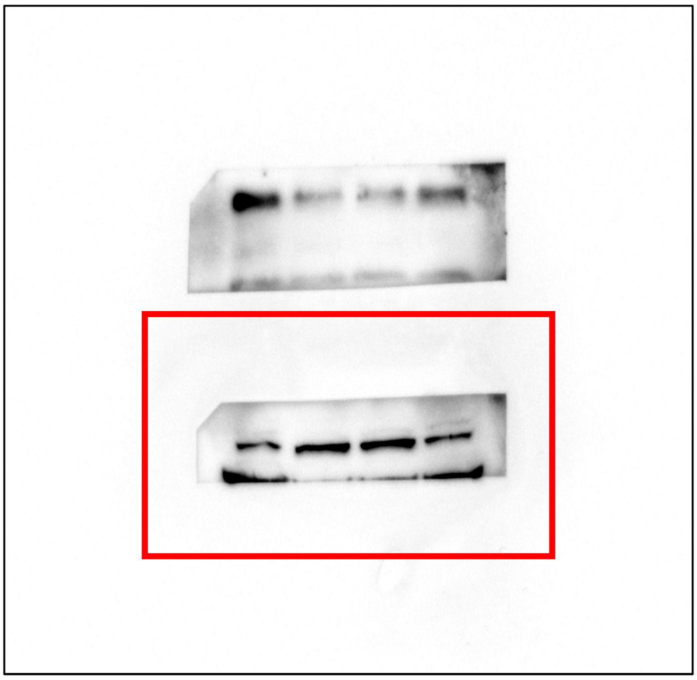

intensity ratio of each band

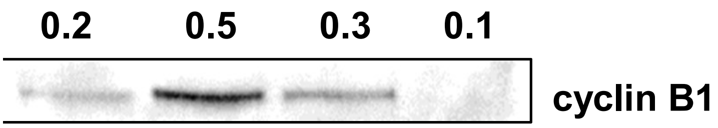

intensity ratio of each band

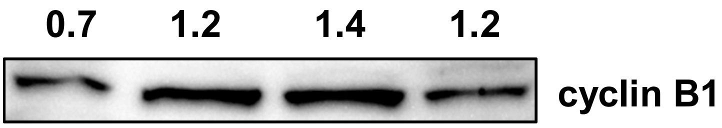

F6

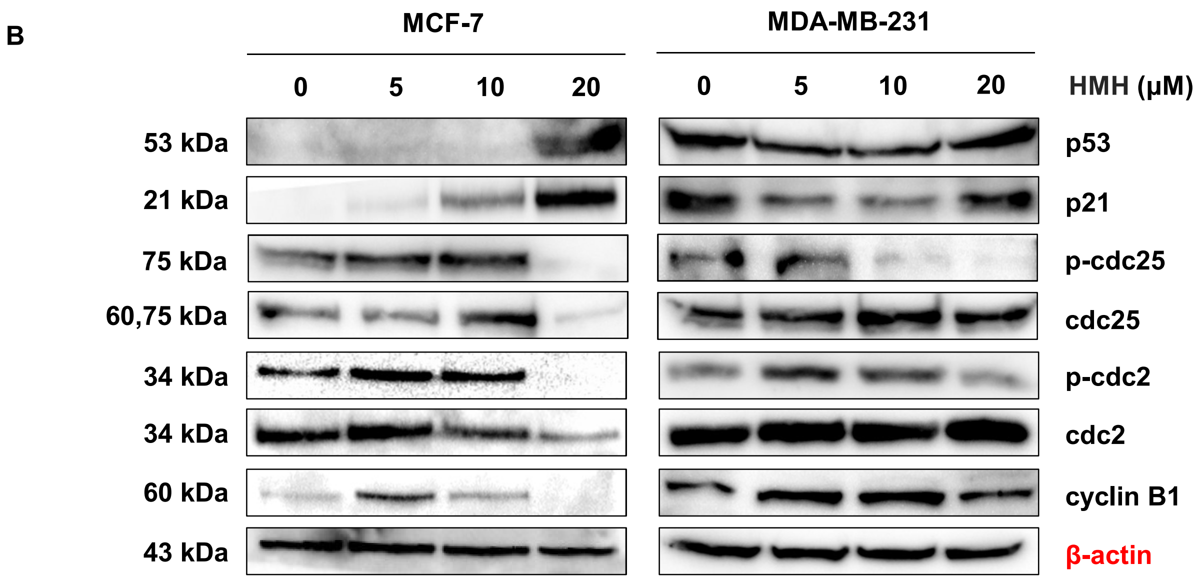
B
MCF-7
MDA-MB-231
0
5
10
20
0
5
10
20
HMH (µM)
53 kDa
21 kDa
75 kDa
60,75 kDa
34 kDa
34 kDa
60 kDa
43 kDa
p53
p21
p-cdc25
cdc25
p-cdc2
cdc2
cyclin B1
β-actin

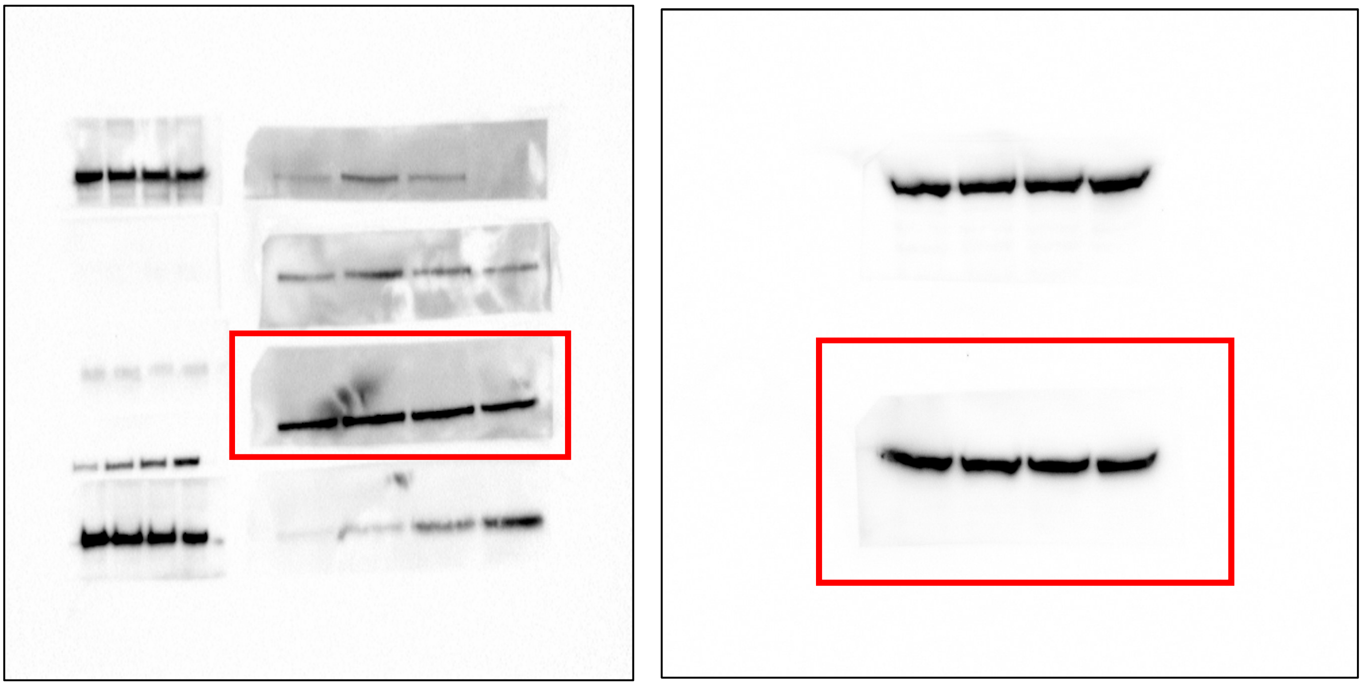

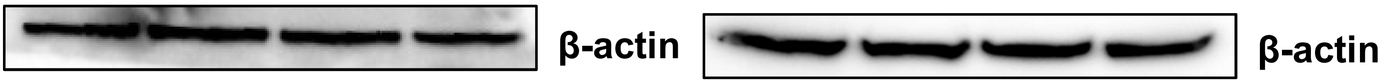
β-actin
β-actin

F6

A

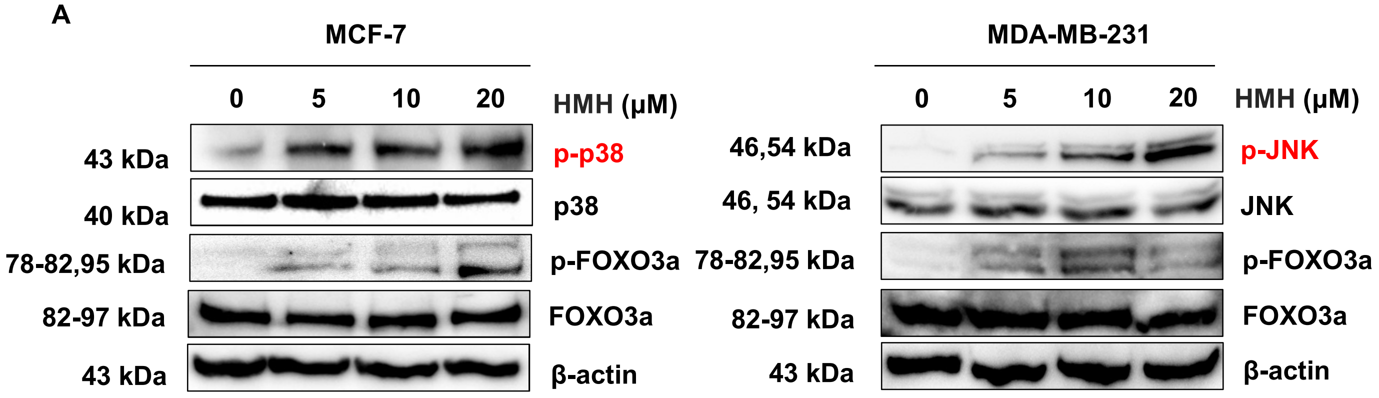

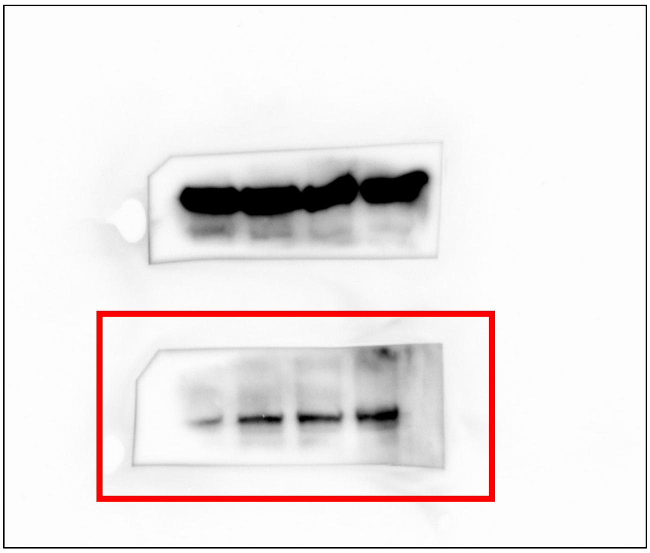

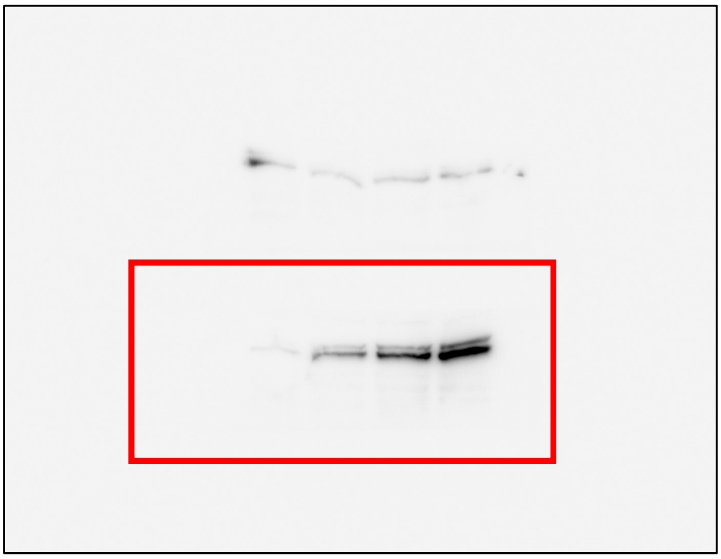

intensity ratio of each band

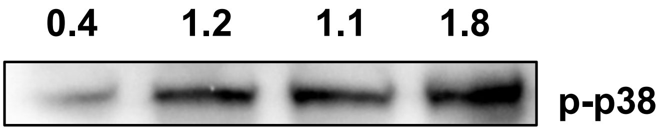

intensity ratio of each band

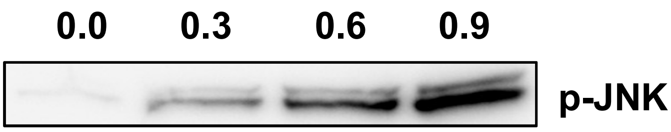

F7

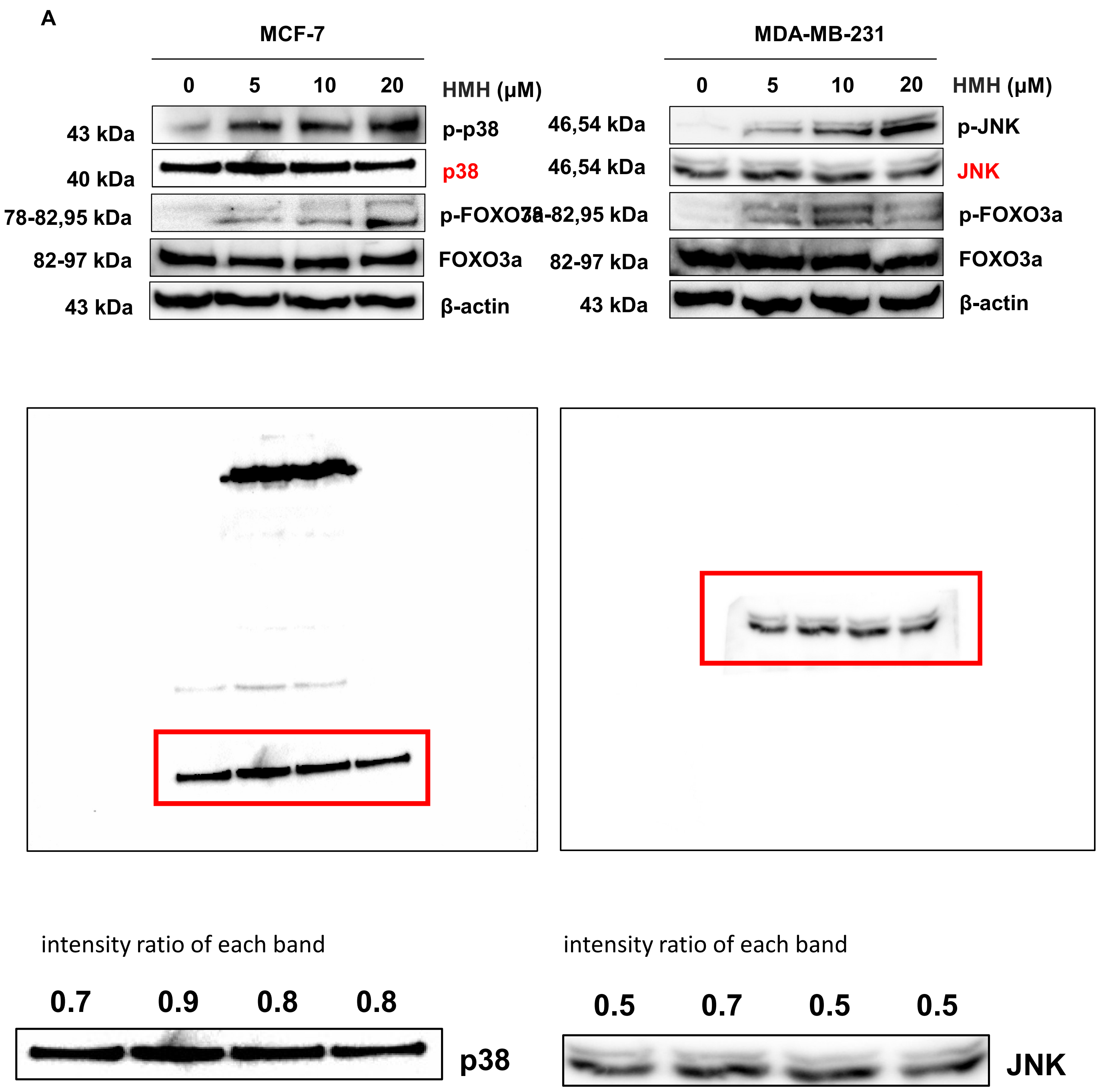

A
MCF-7
0
5
10
20
HMH (μM)
43 kDa
p-p38
40 kDa
p38
78-82,95 kDa
p-FOXO3a
82-97 kDa
FOXO3a
43 kDa
β-actin
MDA-MB-231
0
5
10
20
HMH (μM)
46,54 kDa
p-JNK
46,54 kDa
JNK
78-82,95 kDa
p-FOXO3a
82-97 kDa
FOXO3a
43 kDa
β-actin
intensity ratio of each band
0.7
0.9
0.8
0.8
p38
intensity ratio of each band
0.5
0.7
0.5
0.5
JNK

F7

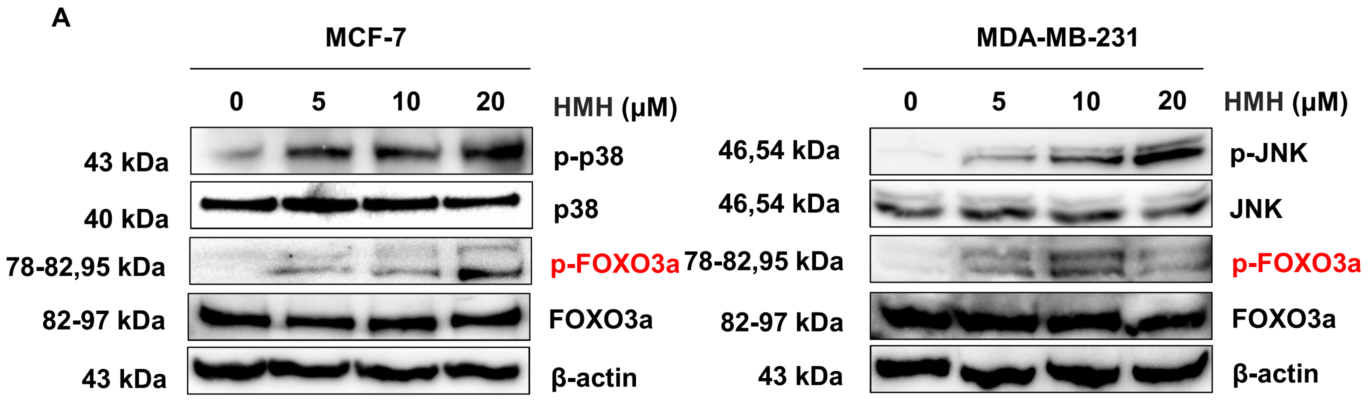
A
MCF-7
0
5
10
20
HMH (μM)
43 kDa
p-p38
40 kDa
p38
78-82,95 kDa
p-FOXO3a
82-97 kDa
FOXO3a
43 kDa
β-actin
MDA-MB-231
0
5
10
20
HMH (μM)
46,54 kDa
p-JNK
46,54 kDa
JNK
78-82,95 kDa
p-FOXO3a
82-97 kDa
FOXO3a
43 kDa
β-actin

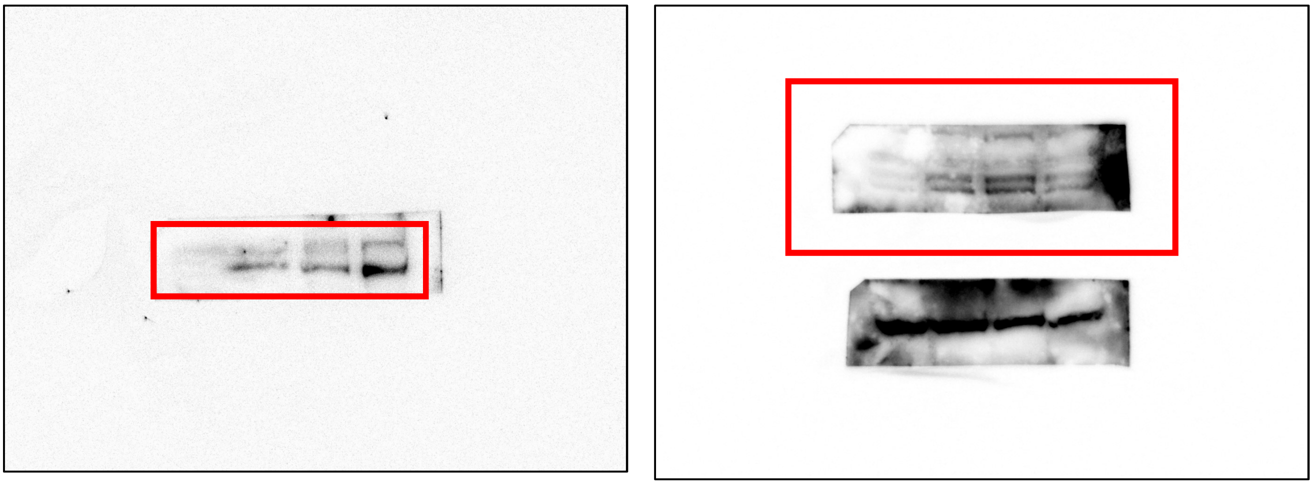

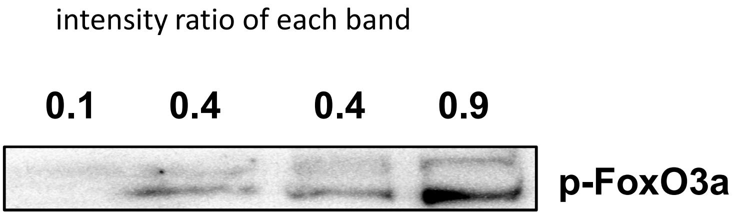
intensity ratio of each band
0.1
0.4
0.4
0.9
p-FoxO3a

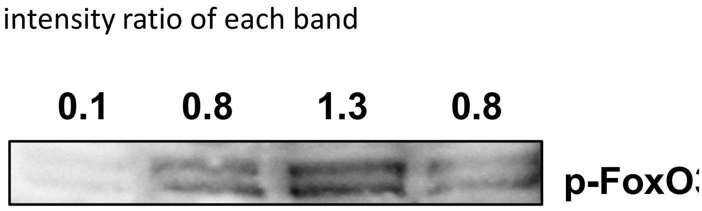
intensity ratio of each band
0.1
0.8
1.3
0.8
p-FoxO

F7

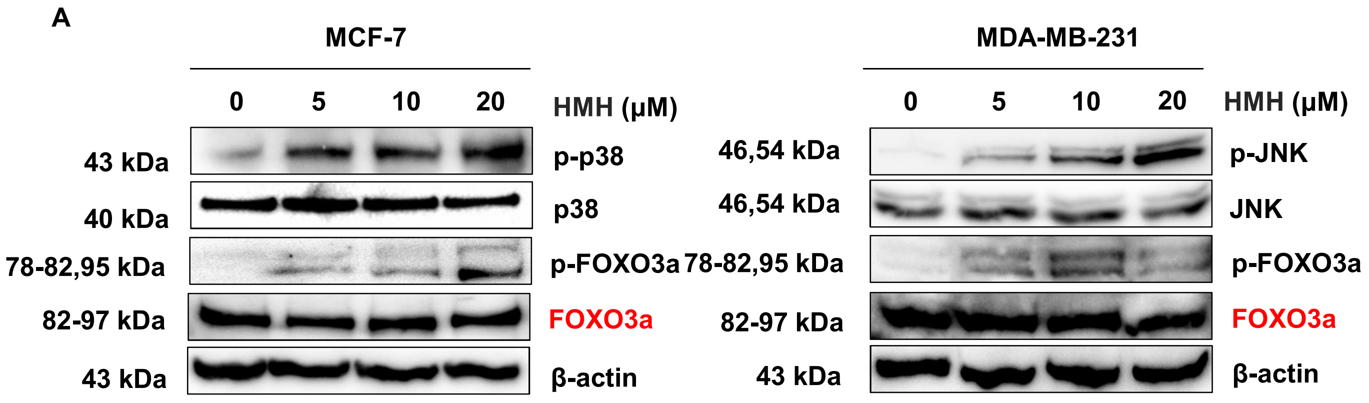
A
MCF-7
0
5
10
20
HMH (μM)
43 kDa
p-p38
40 kDa
p38
78-82,95 kDa
p-FOXO3a
82-97 kDa
FOXO3a
43 kDa
β-actin
MDA-MB-231
0
5
10
20
HMH (μM)
46,54 kDa
p-JNK
46,54 kDa
JNK
78-82,95 kDa
p-FOXO3a
82-97 kDa
FOXO3a
43 kDa
β-actin

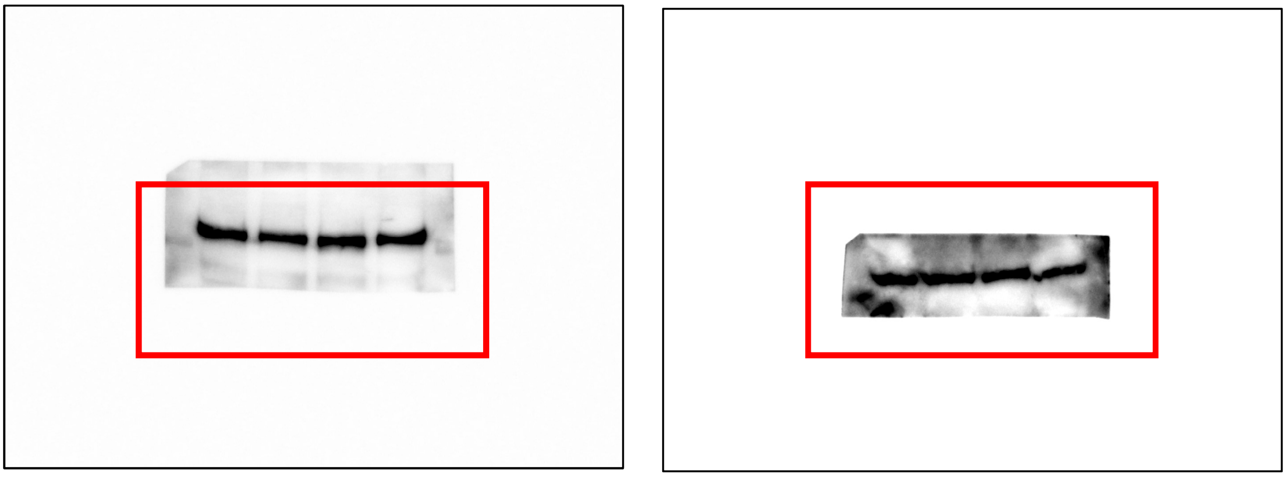

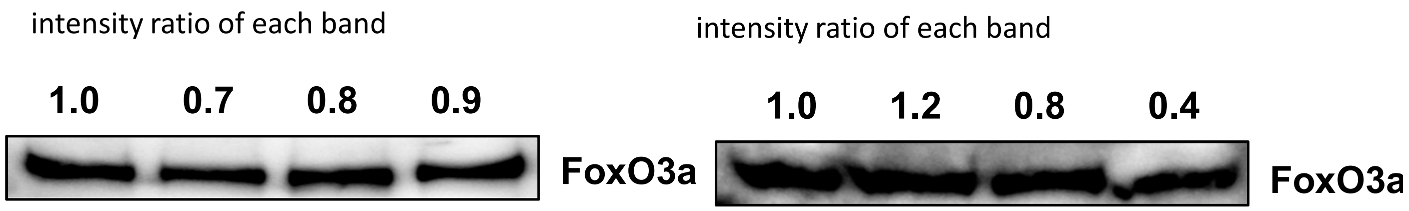
intensity ratio of each band
1.0
0.7
0.8
0.9
FoxO3a
intensity ratio of each band
1.0
1.2
0.8
0.4
FoxO3a

F7

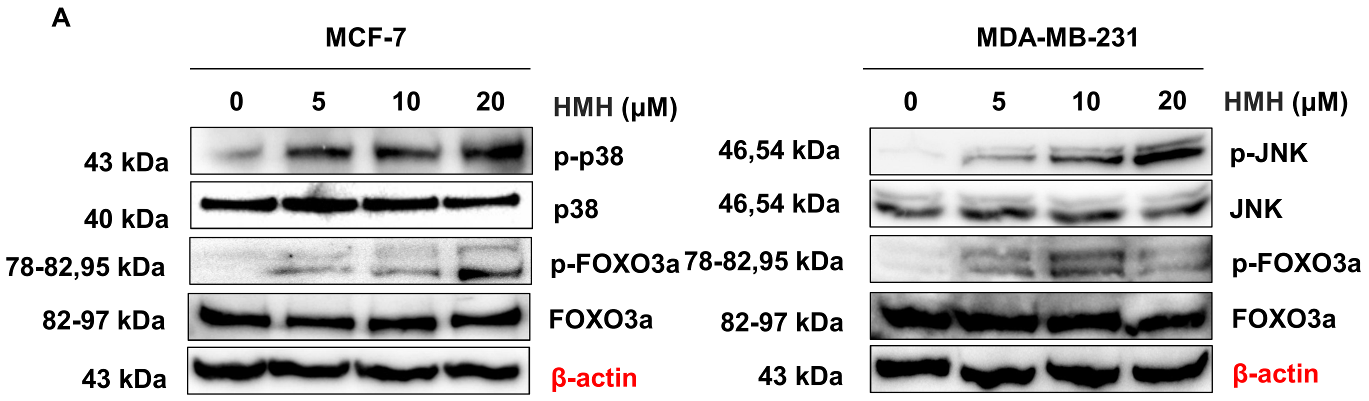

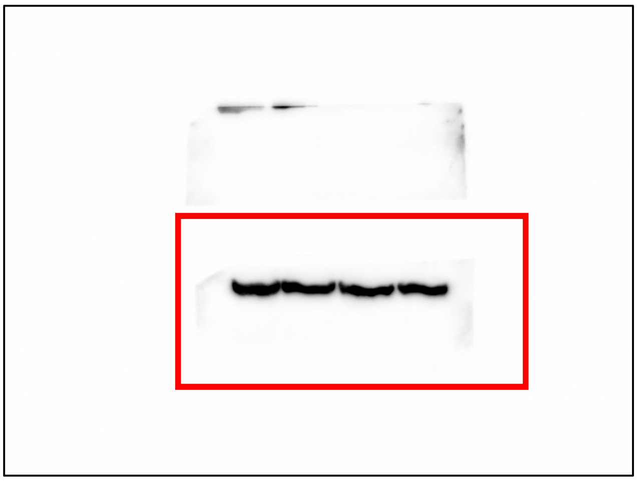

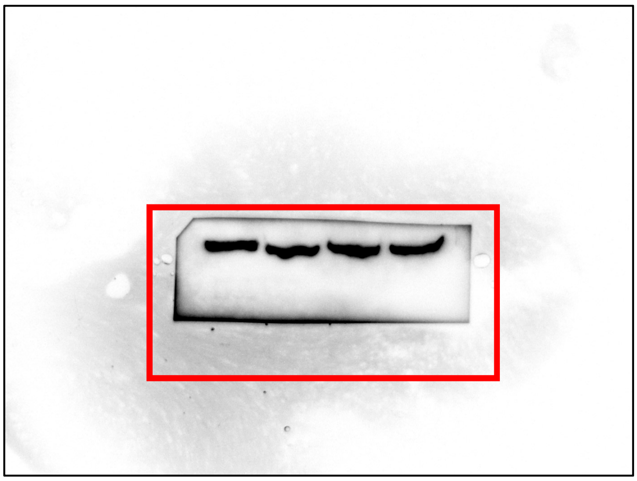

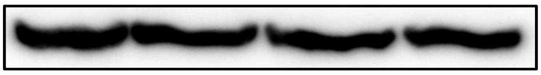

β-actin

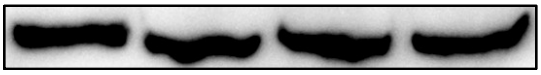

β-actin

F7

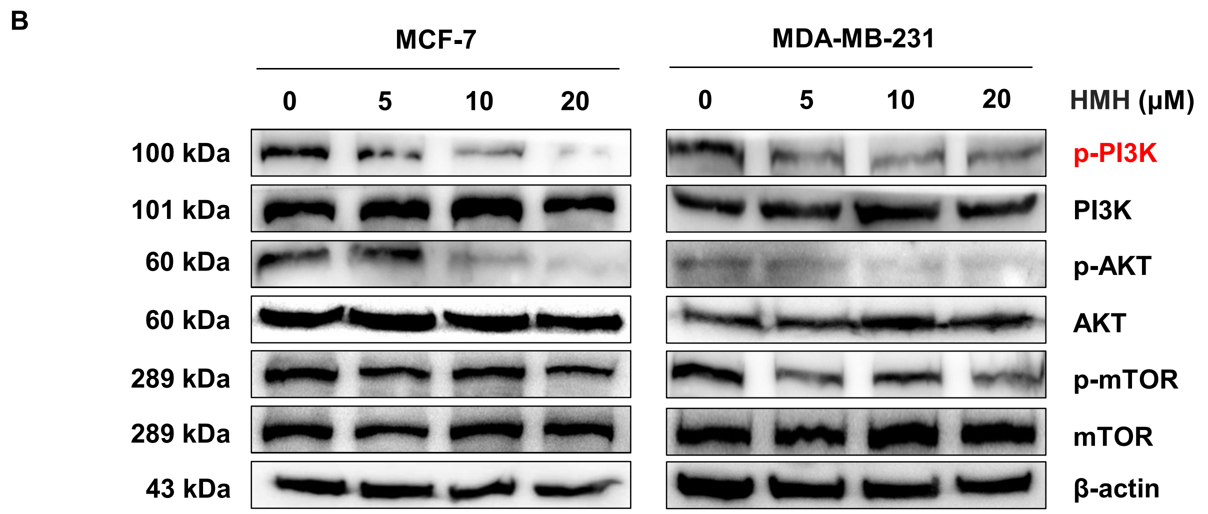

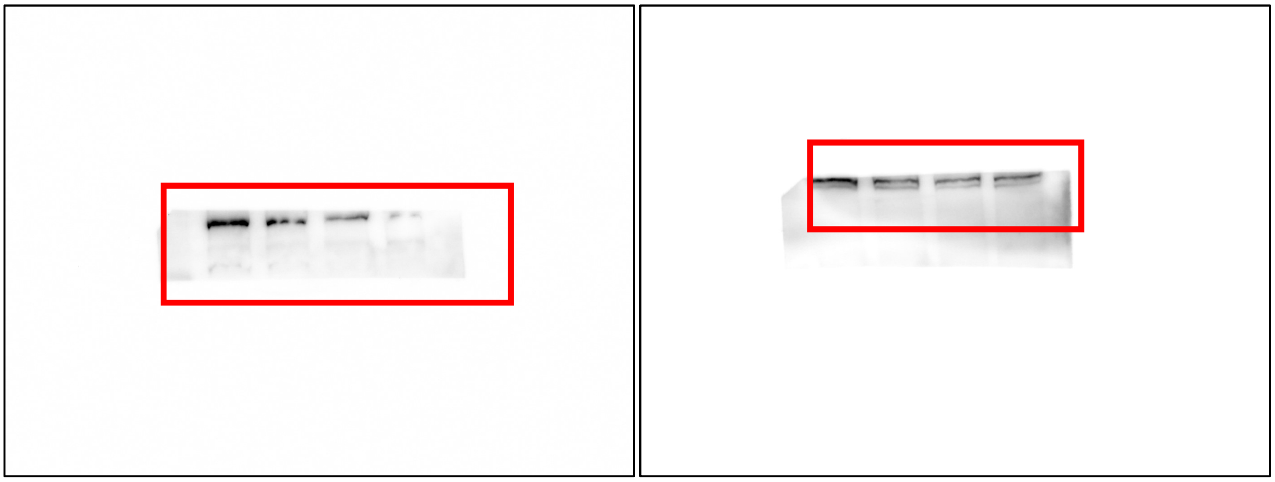

intensity ratio of each band

intensity ratio of each band

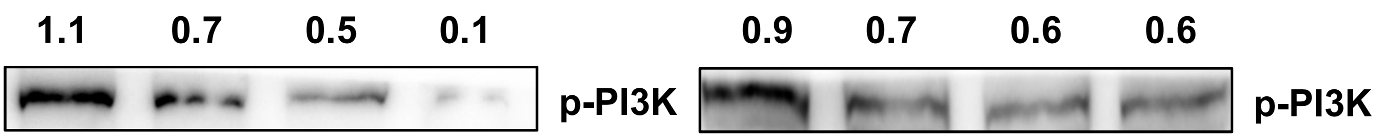

F7

B

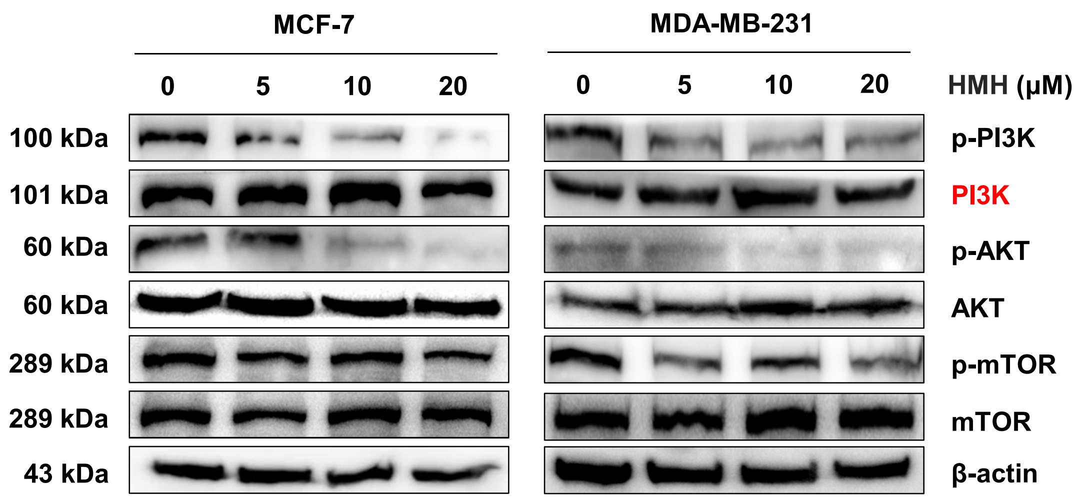

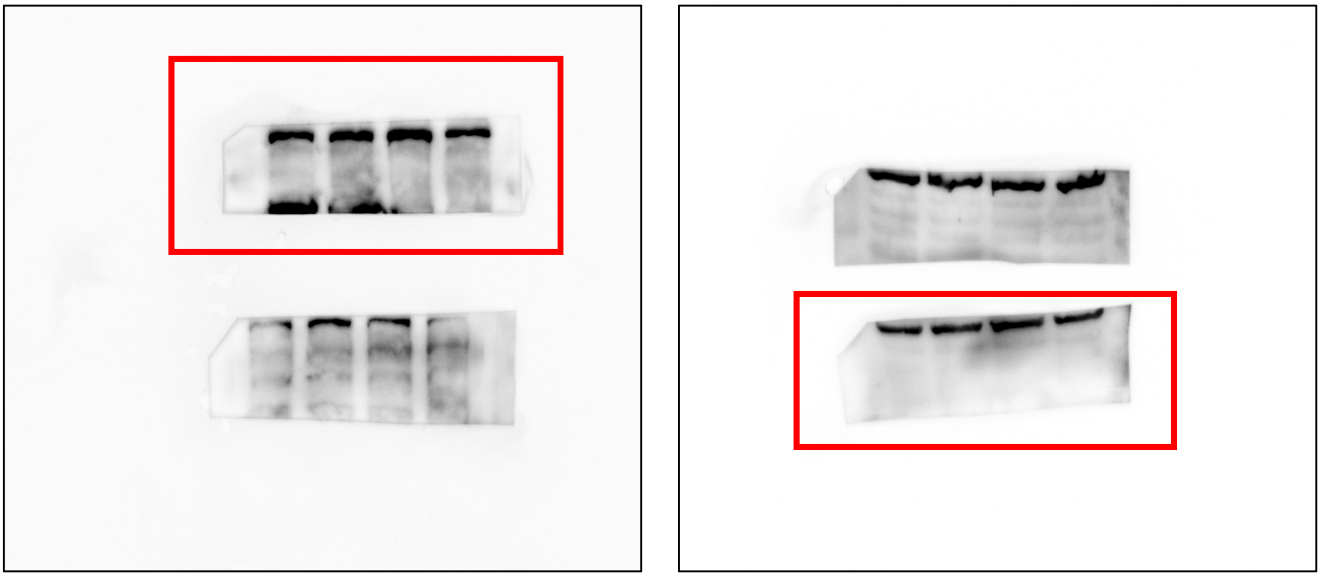

intensity ratio of each band

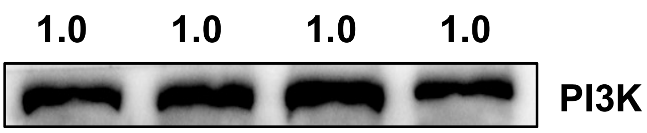

intensity ratio of each band

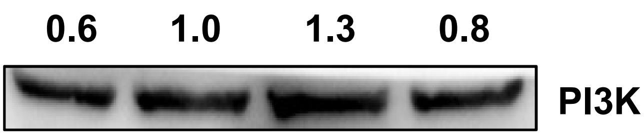

F7

B

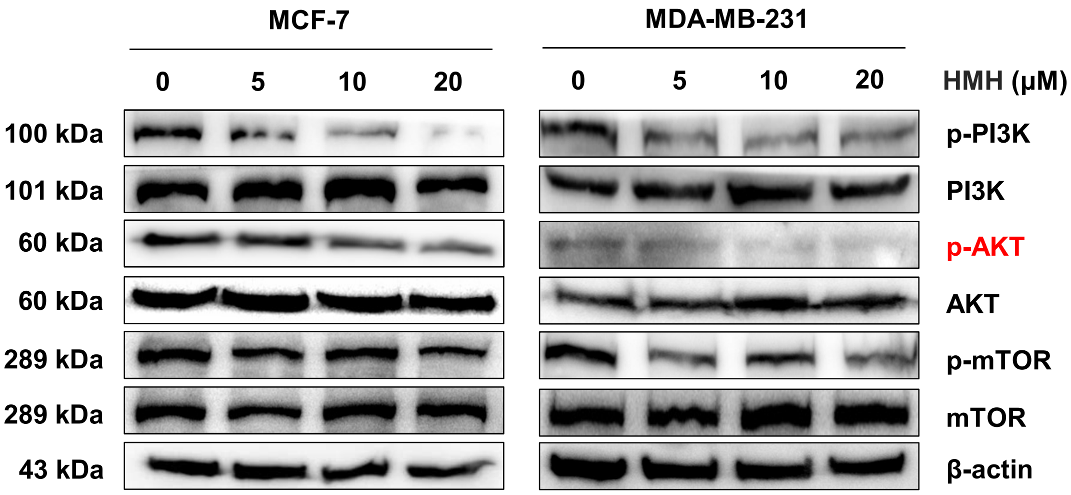

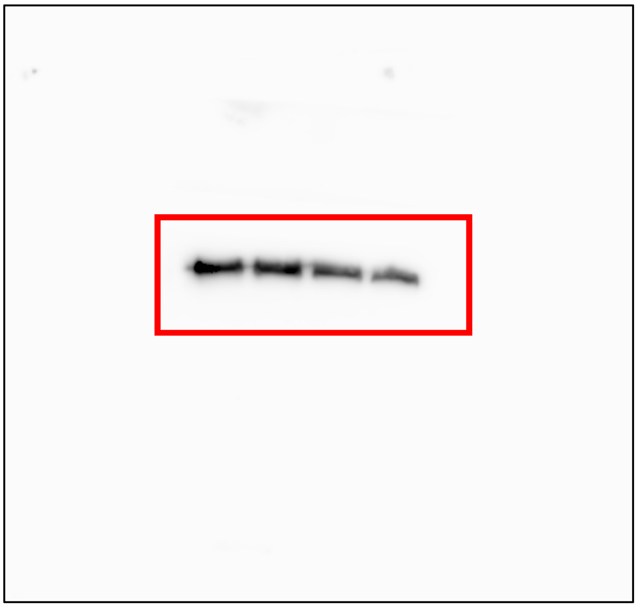

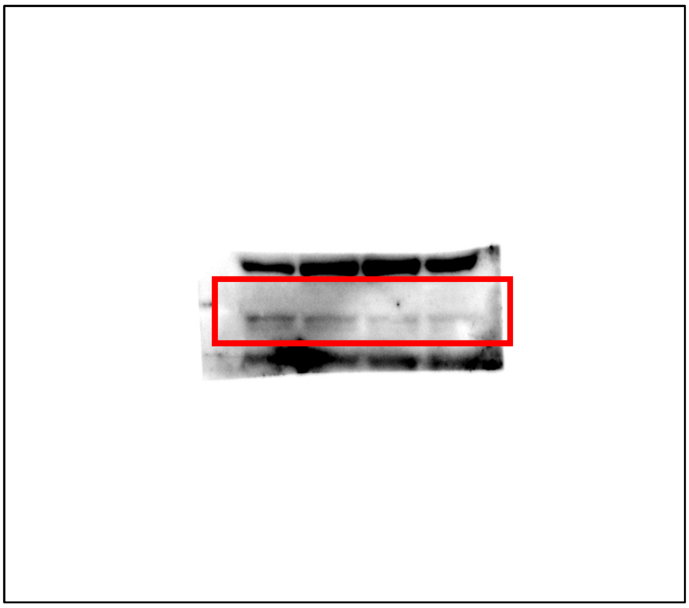

intensity ratio of each band

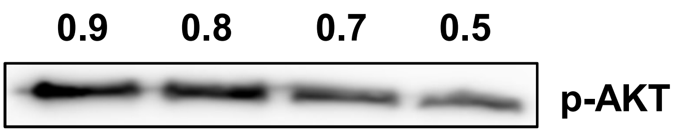

intensity ratio of each band

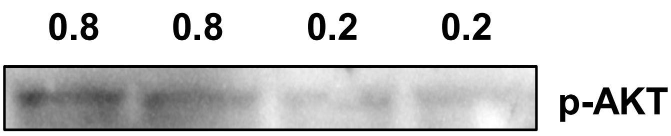

F7

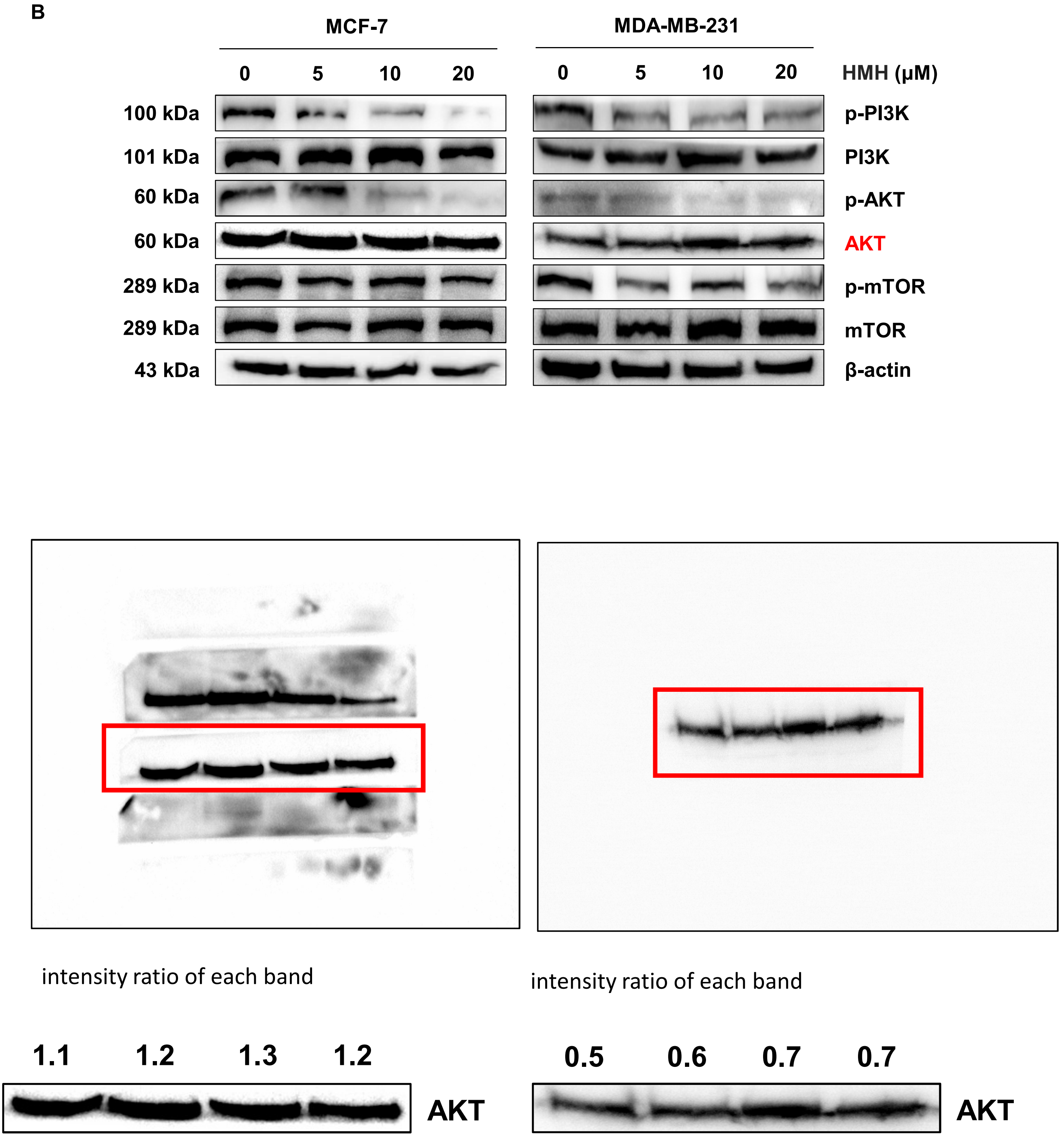
B
MCF-7
MDA-MB-231
0
5
10
20
HMH (μM)
100 kDa
101 kDa
60 kDa
60 kDa
289 kDa
289 kDa
43 kDa
p-PI3K
PI3K
p-AKT
AKT
p-mTOR
mTOR
β-actin
intensity ratio of each band
1.1
1.2
1.3
1.2
AKT
intensity ratio of each band
0.5
0.6
0.7
0.7
AKT

F7

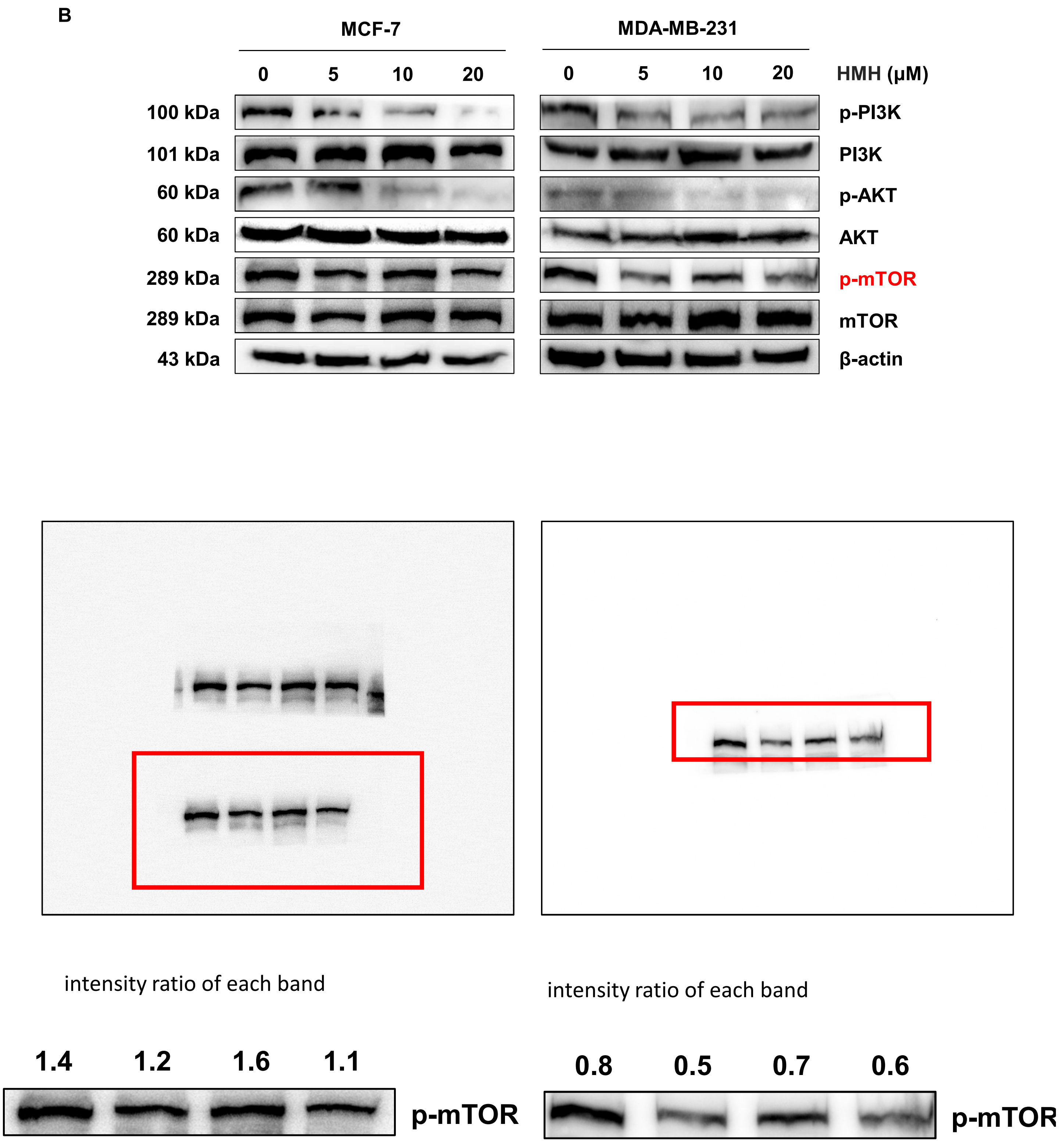
B
MCF-7
MDA-MB-231
0
5
10
20
HMH (μM)
100 kDa
101 kDa
60 kDa
60 kDa
289 kDa
289 kDa
43 kDa
p-PI3K
PI3K
p-AKT
AKT
p-mTOR
mTOR
β-actin
intensity ratio of each band
1.4 1.2 1.6 1.1
p-mTOR
intensity ratio of each band
0.8 0.5 0.7 0.6
p-mTOR

F7

B

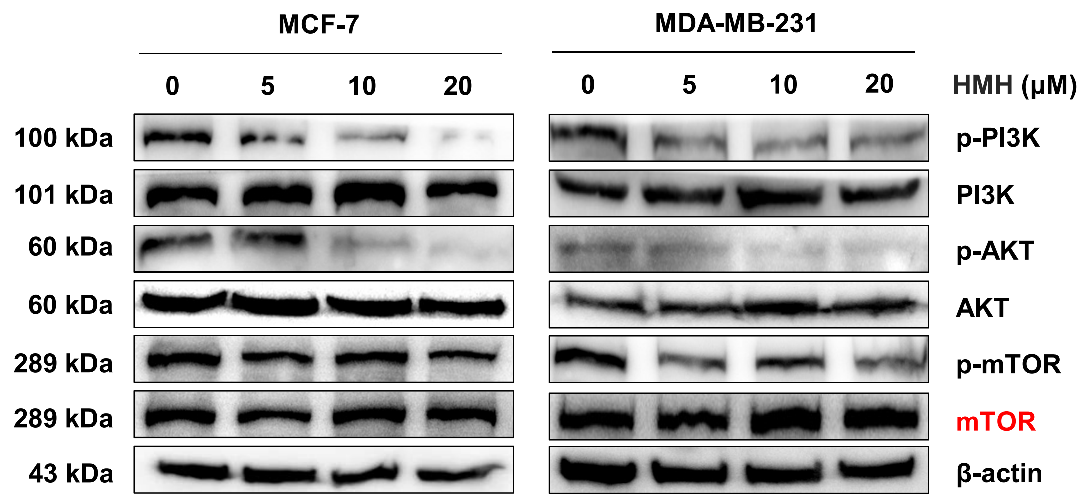

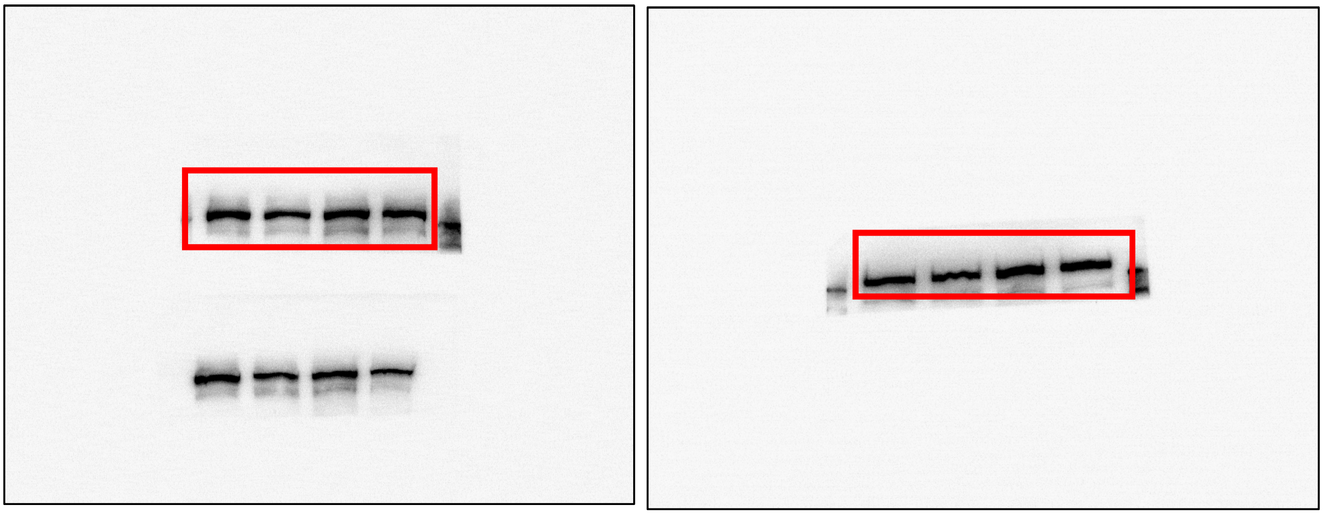

intensity ratio of each band

1.3 0.9 1.5 1.3

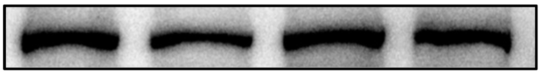

mTOR

intensity ratio of each band

1.1 1.2 1.5 1.4

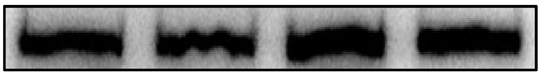

mTOR

F7

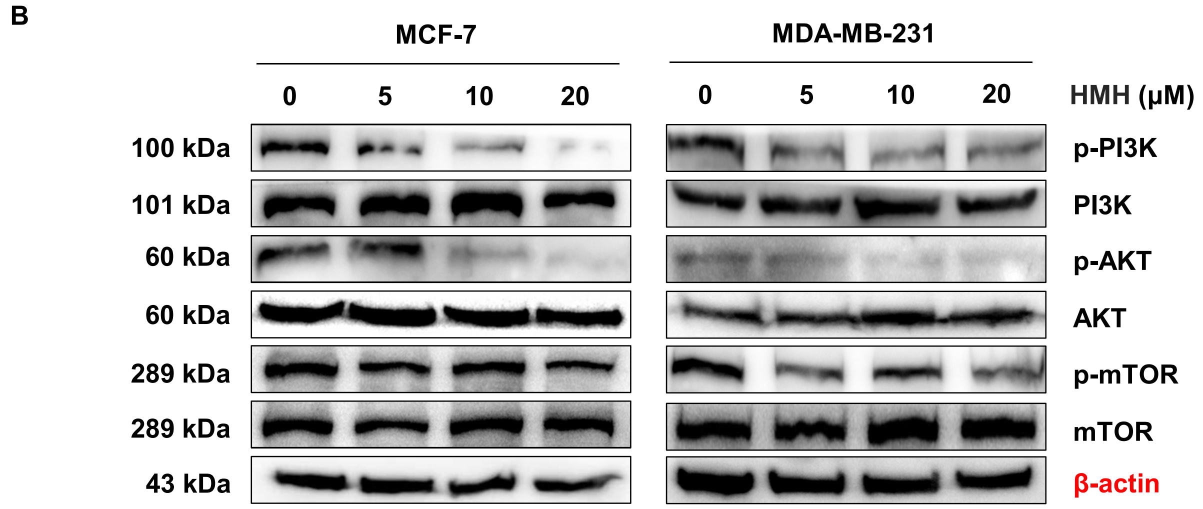

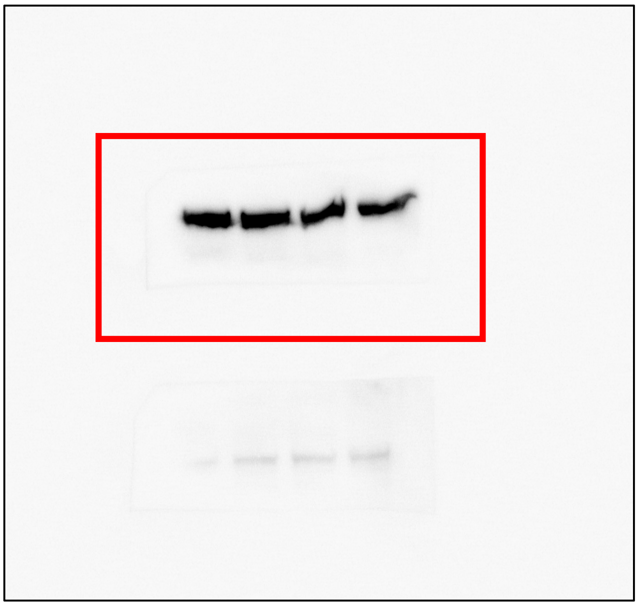

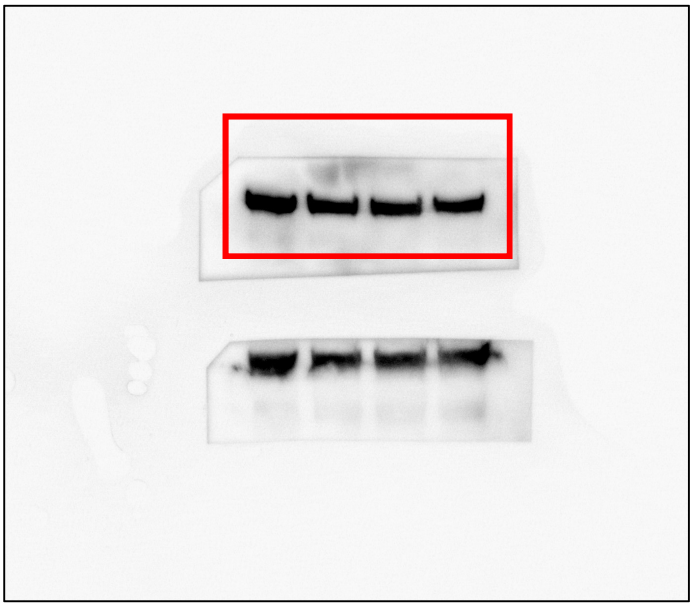

intensity ratio of each band

intensity ratio of each band

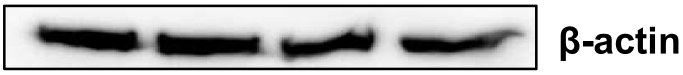

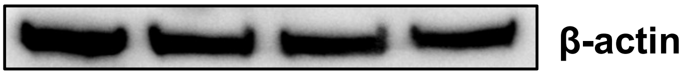

F7

C

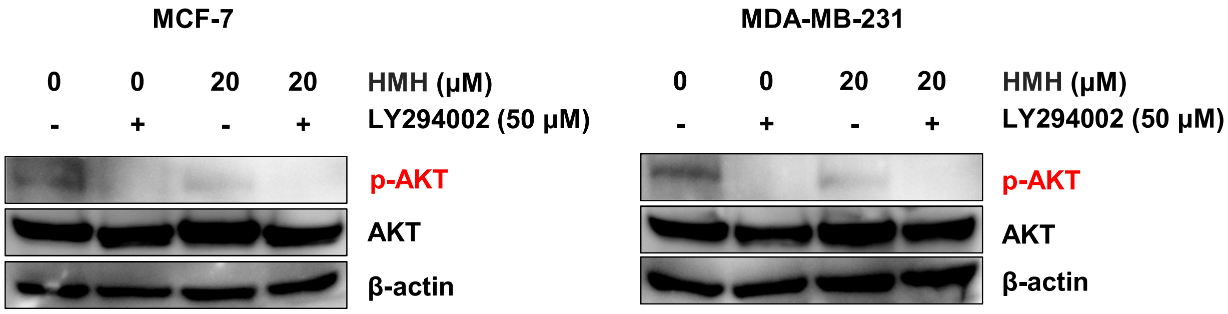
MCF-7
0 0 20 20 HMH (μM)
- + - + LY294002 (50 μM)
p-AKT
AKT
β-actin
MDA-MB-231
0 0 20 20 HMH (μM)
- + - + LY294002 (50 μM)
p-AKT
AKT
β-actin

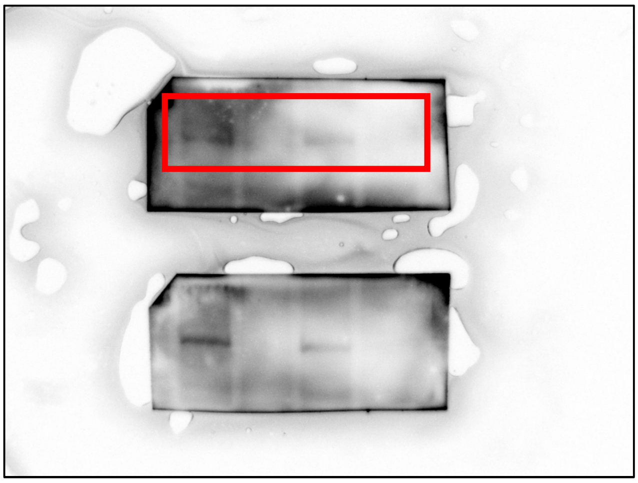

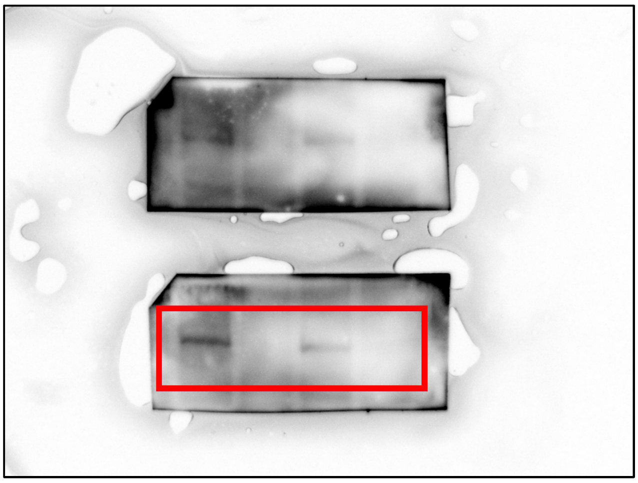

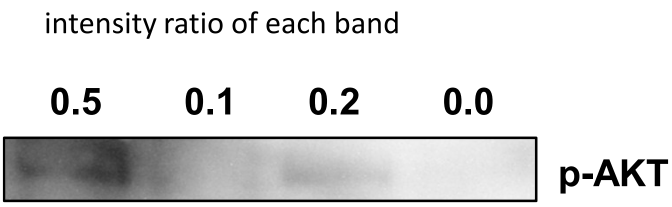
intensity ratio of each band
0.5 0.1 0.2 0.0
p-AKT

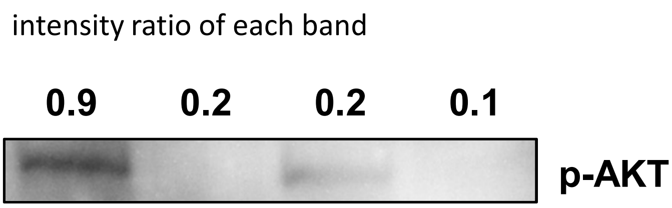
intensity ratio of each band
0.9 0.2 0.2 0.1
p-AKT

F7

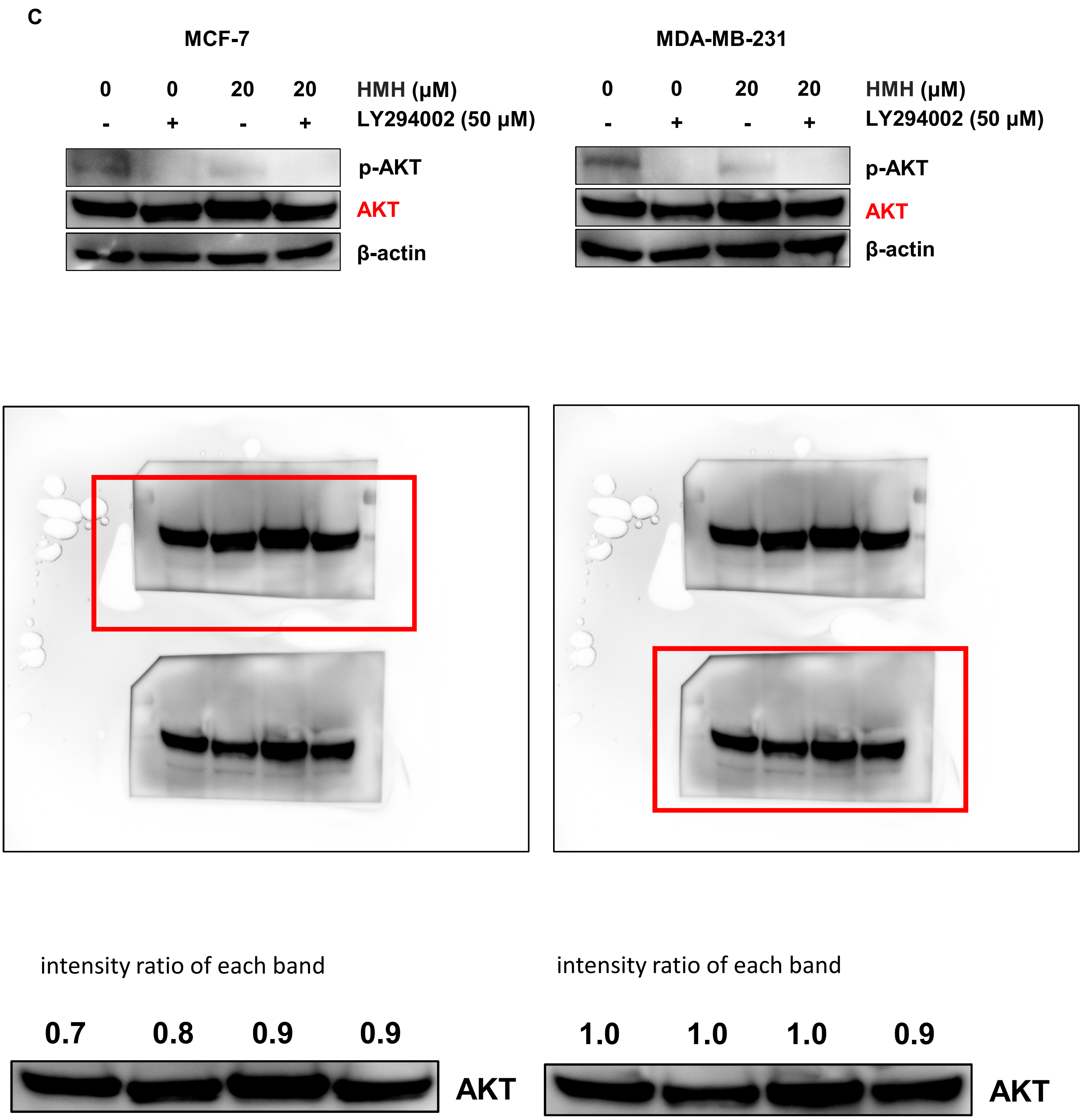
C
MCF-7
0 0 20 20 HMH (µM)
- + - + LY294002 (50 µM)
p-AKT
AKT
β-actin
MDA-MB-231
0 0 20 20 HMH (µM)
- + - + LY294002 (50 µM)
p-AKT
AKT
β-actin
intensity ratio of each band
0.7 0.8 0.9 0.9
AKT
intensity ratio of each band
1.0 1.0 1.0 0.9
AKT

F7

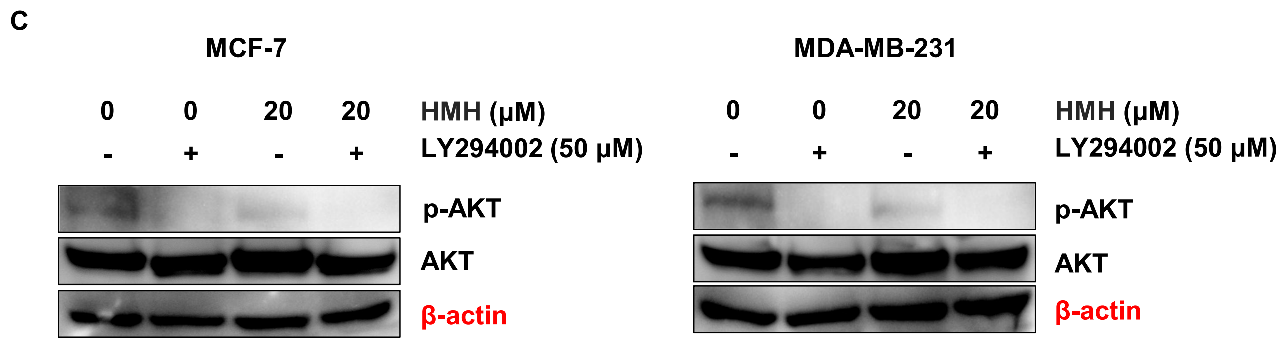

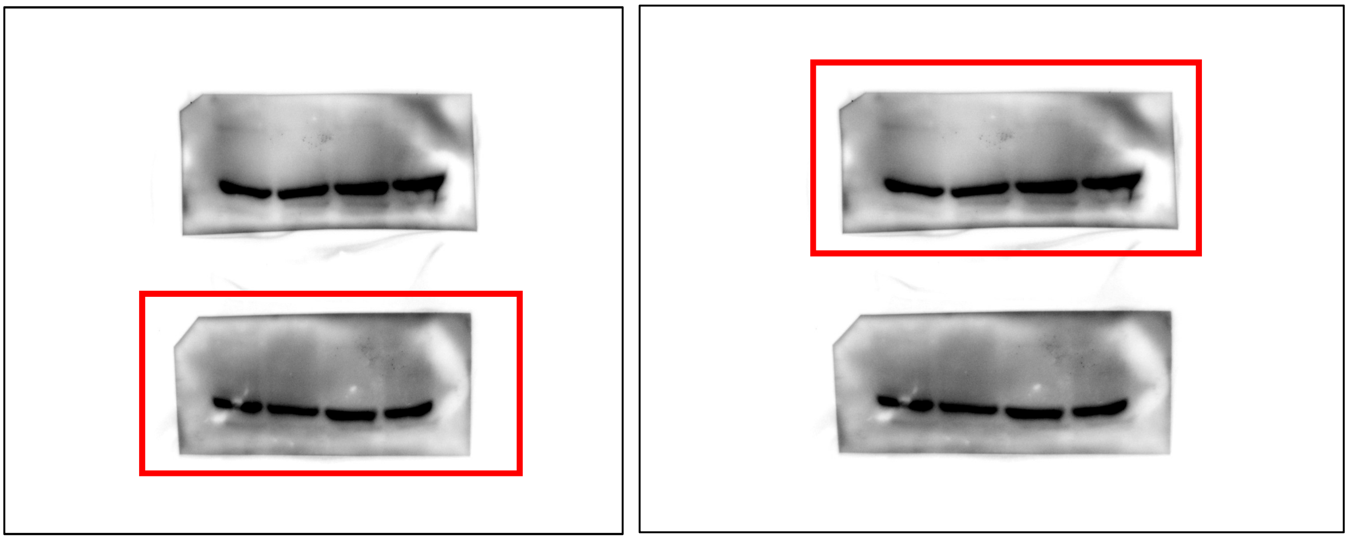

intensity ratio of each band

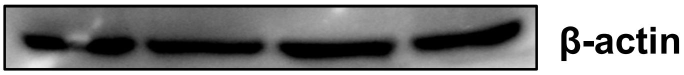

intensity ratio of each band

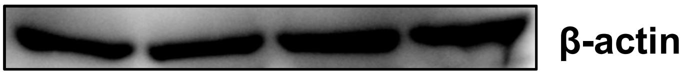

F7
